# From Feedback‐Learning to Semantic Memory: Can Feedback‐Related Brain Activity Predict Object‐Word Associations?

**DOI:** 10.1111/psyp.70306

**Published:** 2026-05-06

**Authors:** Christine Albrecht, Laura Bechtold, Marta Ghio, Christian Bellebaum

**Affiliations:** ^1^ Faculty of Mathematics and Natural Sciences, Institute of Experimental Psychology Heinrich Heine University Düsseldorf Düsseldorf Germany

**Keywords:** feedback learning, FRN, N170, N400, semantic memory

## Abstract

This study investigated the neural mechanisms underlying feedback‐based learning of novel‐object‐novel‐word associations, focusing on how feedback‐locked event‐related potentials acquired during learning relate to subsequent memory performance and acquired association strength. Specifically, we examined whether amplitudes of the feedback‐related negativity (FRN) and N170 components after immediate or delayed feedback predicted not only free recall and recognition performance, but also N400 priming effects exerted by the novel objects on the novel words as a neural correlate of the strength of the associations acquired through feedback‐based learning. Sixty‐six healthy young adults learned novel associations receiving either immediate or delayed deterministic feedback, followed by free recall tests and a newly introduced primed recognition task to measure N400 priming effects. Results showed that FRN amplitudes during learning were associated with recognition performance and predicted frontal N400 priming effects, suggesting a link to procedural, automatically retrieved memories. In contrast, N170 amplitudes were related not only to recognition, but also free recall and to general facilitation of semantic retrieval and integration processes, reflected in reduced N400 amplitudes, indicating a role in declarative memory and familiarity‐driven processing facilitation. Overall, feedback‐based learning elicited robust N400 priming effects, reflecting successful associative integration. However, no consistent effects of feedback timing or valence were observed, likely due to learning strategies adopted based on the deterministic nature of feedback and the anticipation of the memory tasks after learning. These findings highlight distinct contributions of feedback‐related ERP components to different forms of memory representations, linking general mechanisms of feedback‐based learning to the resulting representations.

## Impact Statement

This study directly links prediction‐error‐based learning mechanisms, as indexed by feedback‐locked ERPs, to the formation of semantic memory representations, demonstrating how neural responses during learning directly shape later memory performance and semantic integration, as reflected in N400 priming effects.

The neural mechanisms underlying feedback‐based learning and memory formation in the human brain are still not fully understood, as there seems to be a complex interplay of memory systems and learning mechanisms (Ferbinteanu 2019; Squire 2004). While a basic division of our memory into *procedural*, that is, non‐verbalizable and implicit information, and *declarative*, that is, verbalizable and explicit information, finds wide support (Ferbinteanu 2019), it is a matter of ongoing debate how these different types of memory are acquired (Zárate‐Rochín 2024). One way of acquiring procedural memory seems to be via *feedback learning* (Chase et al. 2011; Gasbarri et al. 2014; Shohamy et al. 2008), which involves the dopaminergic reward system (for a review, see Haber and Knutson 2010). Activity of the striatum, a central structure of the reward system, seems to encode *prediction errors* that drive feedback learning (Carvalheiro et al. 2021; Diederen et al. 2017; Sutton and Barto 2018; Williams et al. 2020), which highlights the role of this type of learning in behavioral adaptation to our ever‐changing environment.

In research using electroencephalography (EEG) in humans, the *feedback‐related negativity* (FRN) in the event‐related potential (ERP) seems to reflect reward system activity (Hajcak et al. 2006; Miltner et al. 1997; Yeung et al. 2005). The FRN is a negative deflection at frontocentral electrode sites around 250 ms post‐feedback onset. Although it might originate from the anterior cingulate cortex (Gehring and Willoughby 2002; Hauser et al. 2014; Nieuwenhuis et al. 2004; Oerlemans et al. 2025), which receives projections from the striatum (Chau et al. 2018), it has been directly linked to striatal activity (Becker et al. 2014; Foti et al. 2011). The FRN is more negative for negative than positive feedback (Gehring and Willoughby 2002; Miltner et al. 1997)^1^ and is further sensitive to prediction error magnitude (Burnside et al. 2019; Fischer and Ullsperger 2013; Weber and Bellebaum 2024).

There is evidence, however, that feedback learning also involves the medial temporal lobe (MTL; Dickerson and Delgado 2015; Dickerson et al. 2011), which is usually associated with *declarative learning* and memory (Squire and Dede 2015; Squire and Zola‐Morgan 1991). This applies especially when feedback is not given immediately: functional magnetic resonance imaging (fMRI) data showed that a delay introduced before the feedback shifts neural activity from the striatum to the MTL (Foerde et al. 2013; Foerde and Shohamy 2011b), which may reflect a shift in learning mechanisms from procedural to declarative (Albrecht et al. 2023). In line with this, increasing feedback delay diminishes FRN amplitude differences between positive and negative feedback, which has been interpreted to reflect a reduced striatal involvement in feedback processing (Höltje and Mecklinger 2020; Peterburs et al. 2016; Weinberg et al. 2012; Weismüller and Bellebaum 2016).

A potential marker of MTL activity, and thus declarative learning, is the N170 (Arbel et al. 2017), a negative‐going temporoparietal ERP component around 170 ms after visual stimulus presentation (see Rossion 2014). The N170 is increased for delayed compared to immediate feedback (Arbel et al. 2017; Höltje and Mecklinger 2020; Kim and Arbel 2019, but see Albrecht et al. 2023 for an opposite pattern). Interestingly, the N170 amplitude also reflects the prediction error magnitude during feedback learning, strengthening its potential role as a marker of (declarative) feedback learning (Röhlinger, Albrecht, and Bellebaum 2025; Röhlinger, Albrecht, Ghio, and Bellebaum 2025).

Only few studies so far have tried to directly link the FRN or the N170 during feedback learning to learning outcomes: Using deterministic feedback, Arbel et al. (2013) and Arbel et al. (2014) found the FRN in response to positive feedback to be more negative for subsequently correctly recognized novel‐object‐novel‐word associations. Höltje and Mecklinger (2020) found generally more negative FRN amplitudes for forgotten than for remembered pictures when these were used as delayed (probabilistic) feedback, while they found no such effect on the N170. Building on Arbel et al. (2017), we used the FRN and N170 in response to deterministic feedback during the learning of novel‐object‐novel‐word associations as predictors of learning success (Albrecht et al. 2023). As the recognition test used by Arbel et al. (2017) can reflect both procedural and declarative learning (Bird 2017; see also Flowers et al. 1984; Isaac and Mayes 1999), we added a free recall test, which reflects presumably declarative MTL‐based learning (Danckert and Craik 2013; Leshikar et al. 2017; Sullivan Giovanello and Verfaellie 2001; Talmi et al. 2015). We found that the N170, but not the FRN, predicts later free recall performance, which supports a specific involvement of the processes underlying the N170 in declarative feedback‐based learning (Albrecht et al. 2023).

The current study first expands the research question how feedback‐locked ERP components during the learning of novel‐object‐novel‐word associations are linked to subsequent memory performance (Albrecht et al. 2023). Notably, in contrast to most previous studies using the subsequent memory paradigm (Mecklinger and Kamp 2023), in the present study we investigated the ERP in response to feedback instead of the to‐be‐remembered items. This allowed the inclusion of feedback‐locked components such as the FRN and the N170, enabling inferences about the effects of feedback‐learning mechanisms. Therefore, we related the FRN and N170 during feedback learning to the participants' free recall and recognition test performance after learning, the latter of which has not been investigated in our previous study (Albrecht et al. 2023). Crucially, we embedded the recognition test into a priming paradigm, allowing us to measure N400 priming effects as a neural correlate of the strength of the associations acquired through feedback‐based learning.

The N400 is a negative deflection in the ERP peaking between 300 and 500 ms after a meaningful stimulus, which is higher for unexpected than expected stimuli (Kutas and Federmeier 2011). In line, the N400 showed a graded amplitude reduction for discrete (Luka and Van Petten 2014) and continuous (Huvermann et al. 2023) manipulations of association strength between known prime and target words (derived from prior knowledge). In novel word learning paradigms, successful learning of novel words is indicated by reduced N400 amplitudes in response to the single words, interpreted to reflect facilitated semantic retrieval (Bakker et al. 2015; Frishkoff et al. 2010). To this date, there is no clear evidence for or against priming effects driven by newly learned associations. On the one hand, there are studies that report no evidence for N400 priming effects when the newly learned word targets are paired with real word primes (Bakker et al. 2015; Korochkina et al. 2024; Liu and van Hell 2020). On the other hand, there is evidence for N400 priming reflecting successfully acquired associations for novel words priming real words (Borovsky et al. 2012, 2013) or pictures (Bermúdez‐Margaretto et al. 2019; Borovsky et al. 2012; Dobel et al. 2010; Korochkina et al. 2024), Further, to our knowledge, it is not known whether feedback‐based learning can be used to form associations with novel words. Notably, investigating the N400 as a fine‐grained marker of successful feedback‐based learning allows us to relate the varying strength of associations to distinct electrophysiological markers of learning.

As our second and main research question, we were thus interested to see whether novel‐object‐novel‐word associations learned via feedback elicit an N400 priming effect on novel word processing and, importantly, whether the strength of this effect can be predicted by feedback‐related ERP components, that is, the FRN or N170. Further, the N400 has been interpreted as a prediction‐error based learning signal in the context of semantic processing (Rabovsky and McRae 2014; Fitz and Chang 2019), which connects the N400 to the FRN and N170 during learning. The novelty of this study therefore lies in directly linking general mechanisms of prediction‐error based learning (Nixon 2020) to the resulting semantic memory representations.

We hypothesized that better memory performance (i.e., higher recognition and free recall accuracy) should relate to ERP amplitudes during learning. In accordance with our previous study (Albrecht et al. 2023), we expected the N170, but not the FRN, to predict later free recall performance, with lower amplitudes indicating successful memory performance (Röhlinger, Albrecht, and Bellebaum 2025; Röhlinger, Albrecht, Ghio, and Bellebaum 2025). This effect should depend on feedback timing (being more pronounced for delayed feedback), which we manipulated between‐subjects. Both components, FRN and N170, might predict later recognition performance—the FRN more for associations learned with immediate, the N170 more for associations learned with delayed feedback.

Regarding the N400 priming effects as marker of acquired association strength, we hypothesized that objects that were associated with specific words during the feedback learning task can serve as primes for these words after learning, leading to reduced N400 amplitudes compared to unrelated novel‐object‐novel‐word pairs. Regarding our main research question, we expected that higher association strength (i.e., stronger N400 priming effects) should be predicted by ERP amplitudes during feedback learning. For the N400, as for recognition performance, we expected that both the FRN and N170 during learning might predict the association strength as measured by the N400 amplitude reduction, with a potential role of feedback delay: the FRN again more for associations learned with immediate, the N170 more for associations learned with delayed feedback.

## 1 Method

### 1.1 Participants

After we found robust results in a sample size of 28 participants in a previous study with feedback delay as a within‐subject factor (Albrecht et al. 2023; Power = 81.3%) for an effect of N170 amplitude on free recall performance, we aimed for at least twice as many participants for this study, in which feedback delay was included as a between‐subject factor. Taking a potential drop‐out rate of 20% into account, we recruited 72 participants. Of those, a total of six participants were excluded, either due to technical issues during acquisition (*n* = 2), extensive EEG signal artifacts (*n* = 2), or alpha activity during the experiment (*n* = 2). All participants were right‐handed, had normal or corrected‐to‐normal vision, and reported no neurological or psychological medical history. Additionally, none of the participants reported being dyslexic or taking medication affecting the central nervous system. Of the 66 participants that entered the analysis, 33 participants (19–39 years, *M* = 25.1 years, SD = 4.5 years, 20 cis‐gender women, 12 cis‐gender men, 1 trans‐gender man) learned with immediate feedback and 33 (18–38 years old, *M* = 23.7 years, SD = 4.2 years, 21 cis‐gender women, 12 cis‐gender men) with delayed feedback.

### 1.2 Stimuli

**FIGURE 1:**
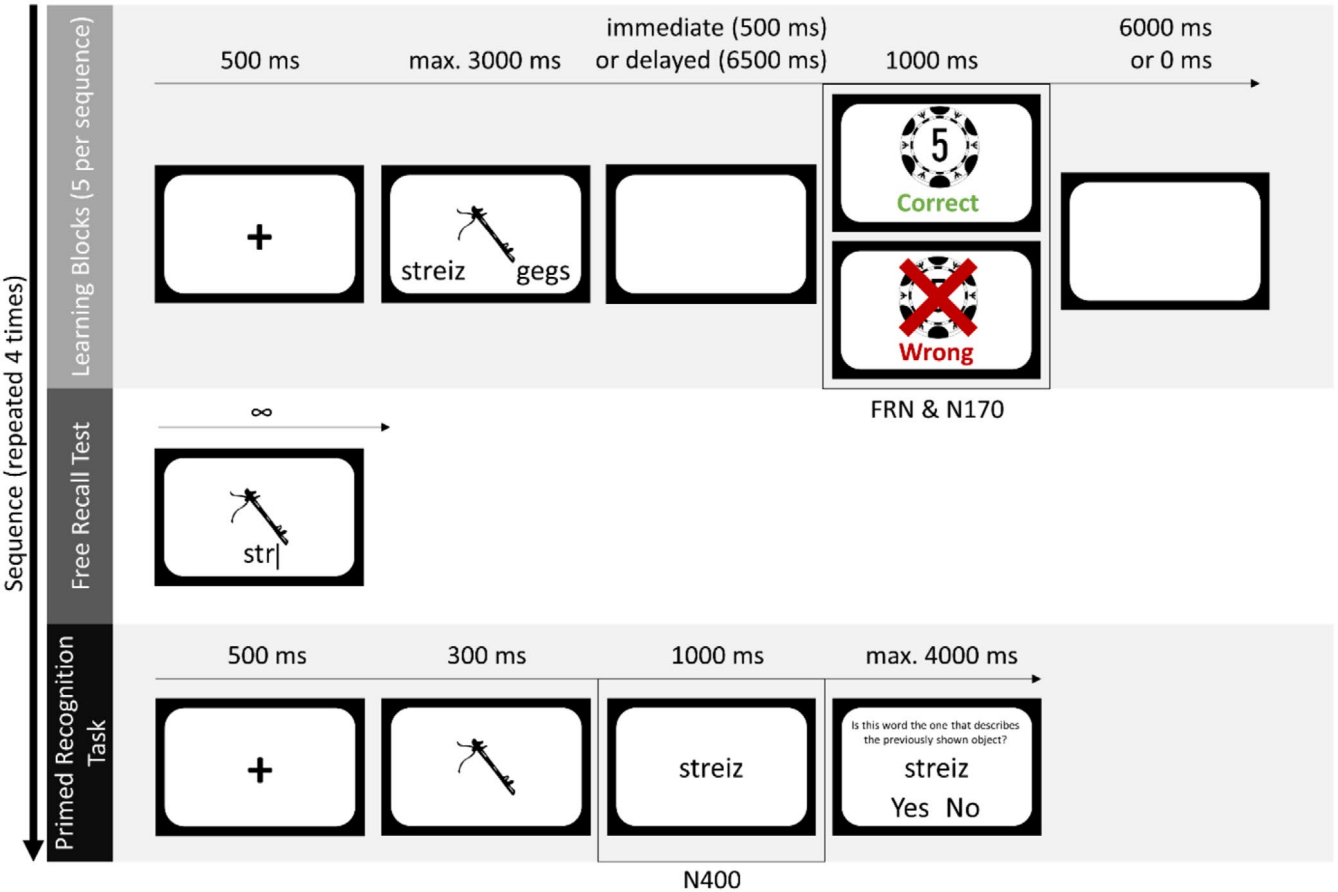
Trial structure in the learning blocks, free recall test and primed recognition task. A new set of 14 novel‐object‐novel‐word combinations was used for each sequence. The FRN and N170 were event‐locked to the feedback presentation during the learning blocks. The N400 was event‐locked to the novel word presentation during the primed recognition task.

As in the preceding study (Albrecht et al. 2023), participants learned associations between 56 novel objects and 56 respective novel words (see Figure 1 for examples). Further, 56 novel words served as distractors. The stimuli were adopted from Albrecht et al. (2023), who respectively adopted the novel objects from Kroll and Potter (1984) and created a set of 112 novel words suitable for German native speakers. In the current study, the novel objects were further transformed to vector graphics (using Adobe Illustrator 2023, Adobe Inc., 2019) to improve image resolution. Two of the novel words were randomly assigned to each of the novel objects; one was selected as the target and one as the distractor (based on the performance in the first block, see below). The 56 combinations of novel object, target word, and distractor were subsequently sorted into four sets of 14 combinations each.

### 1.3 Experimental Task

The experimental task was nearly identical to Albrecht et al. (2023; see also Arbel et al. 2017 for the original paradigm), with two changes: First, the recognition test was changed to a primed recognition task; second, the feedback delay was manipulated in a between‐subject design instead of a within‐subject design.

The study included four sequences, each of which used one of the predetermined sets of novel objects and novel words. In each sequence, the participants could thus learn associations between 14 novel objects and the respective novel words. Each sequence started with five feedback learning blocks, followed by a free recall test and ending with the primed recognition task.

#### 1.3.1 Feedback Learning Blocks

All 14 novel‐object‐novel‐words‐combinations per sequence were presented once per learning block in random order. Participants were instructed to learn which of the two novel words presented on the left and right side of a computer screen was associated with the given novel object that was presented simultaneously. For this learning of the respective target word, they were instructed to choose one of the novel words per trial by pressing the left or right button marked on a USB keyboard. A novel object was always presented with the same two novel words, whose positions on the screen were randomly determined (target left, distractor right, or vice versa). After choosing one novel word, participants received feedback with 100% contingency, that is, always positive feedback when the target was chosen and negative when the distractor was chosen, respectively. In the first learning block of each set, participants were randomly given positive feedback in exactly 50% of trials and negative feedback in the other 50%. Depending on their choice, the chosen word was then set as the target word (for positive feedback) or distractor word (for negative feedback) for all following learning blocks and tests. As in Albrecht et al. (2023), we aimed to improve motivation by having participants win fictional casino chips for each correct response, allowing them to beat a fictional high score. For each correct response in the learning blocks, participants received one casino chip with a 5‐point value. Participants were informed that the received casino chips had no impact on their compensation.

A trial in a learning block (see Figure 1, upper part) included a 500 ms fixation cross, followed by the novel object displayed at the center of the screen, with two novel words below, one on the left and one on the right side. Participants had a maximum of 3000 ms to choose one of the novel words by pressing the respective button. Before the start of the experiment, participants were randomly assigned to either the immediate feedback condition or the delayed feedback condition. In the immediate feedback condition, participants saw a white screen for 500 ms after the choice. In the delayed feedback condition, participants received feedback 6500 ms after their choice. If the target novel word was chosen, feedback was positive (a picture of a casino chip worth five points and the word “correct” in green font). If the distractor novel word was chosen, feedback was negative (the same casino chip, but crossed out, and the word “wrong” in red font). If participants did not respond within 3000 ms, they did not receive feedback, but saw a picture of a snail with the words “too slow!” always 500 ms after the time window ended (in both feedback delay groups). Trials with responses exceeding the response deadline were not repeated and accounted for a negligible proportion of trials (*M* = 0.85%, SD = 0.81%, ranging from 0% to 3.75% per participant). Feedback and miss indications were each presented for 1000 ms. In order to make the two versions equally long, an inter‐trial interval of 6000 ms (empty screen) followed the feedback in the immediate feedback group. After the completion of each learning block, participants were shown a bar visualizing their progress in the current learning session as well as their current score.

#### 1.3.2 Free Recall Test

After the five feedback learning blocks per sequence were completed, participants underwent a free recall test. In the test (see Figure 1, middle part), all the 14 novel objects were displayed once in random order on the computer screen and participants were instructed to type the target word using the keyboard. In each trial, the novel object was displayed at the center of the screen and the entered text was displayed below. Participants had unlimited time to type their answer, and were told to submit the answer using the Enter key. Participants did not receive feedback in the free recall test. However, they were told before the test that they would receive 10 casino points for each correct answer, and they were shown a bar visualizing their progress in the current learning session and their current score after the completion of the test.

#### 1.3.3 Primed Recognition Task

Instead of the recognition test used in Albrecht et al. (2023), we implemented a primed recognition task, which allowed us to assess event‐related potentials in response to the novel word. In this task, each novel object was shown four times, followed by either the target word (associated target), the distractor word for the novel object (associated distractor), the target word of another novel object in the same block (not associated target), or the distractor word of another novel object in the block (not associated distractor). In total, the primed recognition task consisted of 56 trials for each set (i.e., 224 overall), 14 (i.e., 56 overall) each for the associated target, associated distractor, not associated target and not associated distractor condition.

Each trial (see Figure 1, lower part) started with a 500 ms fixation cross. Subsequently, a novel object was displayed for 300 ms at the center of the screen. Next, a novel word appeared on the center of the screen for 1000 ms. Following this, participants saw the question “Is this word the one that describes the previously shown object?” and had a maximum of 4000 ms to press the corresponding button to “yes” or “no.” The two response options were displayed on the left and right side on the bottom of the screen, with randomized positions (“yes” left and “no” right or vice versa) to prevent participants from conducting or preparing their response already while the novel word was displayed. Participants did not receive feedback for their choice, but again, they were told before the task that they would receive 10 casino points for each correct response, and a progress bar and their current score were shown to them at the end of the task. If participants failed to respond in the time window of 4000 ms, they were shown the same miss indication as in the learning blocks, also for 1000 ms. Trials with responses exceeding the response deadline were not repeated. They accounted for a negligible proportion of trials (*M* = 0.16%, SD = 0.56%, ranging from 0% to 4.02% per participant).

### 1.4 Procedure

Participants were alternatingly assigned to the immediate or delayed feedback version of the experiment upon arrival in the laboratory for the single subject testing session. Before the experiment, participants gave their informed written consent to participation and filled out the demographic questionnaire. The experimenters then attached the EEG electrodes and started the experiment. Subsequently, the experimenters went carefully through the written instructions with the participants, reading out the standardized instructions presented on screen and giving the opportunity for further questions. If the participants were confident that they understood the task, EEG recording began, and participants engaged in four experimental sequences, each consisting of five consecutive learning blocks followed by one free recall test and one primed recognition task. Throughout the experiment, participants could pace the instructions and the start of each block/test themselves and were instructed to take breaks on these occasions if they wished. At the end of the experiment, participants were shown a fictitious leader board including their own score as part of the overall motivation instruction (which was to beat the fictional high score). The experiment lasted between 60 and 90 min, and together with the questionnaire and EEG preparation, participants spent about 2–2.5 h in the lab. Participants were reimbursed monetarily or with course credit.

### 1.5 EEG Recording

A 32‐channel textile ActiCap electrode cap (Brain Products GmbH, Germany) and the software Brain Vision Recorder (version 1.20; Brain Products, Munich, Germany) were used to record EEG signals at a 1000 Hz sampling rate with active silver/silver‐chloride electrodes. The electrodes were attached on 29 scalp sites according to the extended 10–20 system: FCz (used as online reference), F7, F3, Fz, F4, F8, FC5, FC1, FC2, FC6, T7, C3, Cz, C4, T8, CP5, CP1, CP2, CP6, P7, P3, Pz, P4, P8, PO9, P1, Pz, P2, and PO10. Additionally, two electrodes were placed on the bilateral mastoids for offline re‐referencing, and vertical electrooculogram (vEOG) was measured at FP2 and below the right eye to monitor eye blinks. All impedances were kept below 15 kΩ.

### 1.6 EEG Data Preprocessing

We preprocessed EEG data with the Brain Vision Analyzer software (Brain Products, Munich, Germany). The data were first re‐referenced to the average of all scalp electrodes (excluding the mastoids and vEOG electrodes) and the data at the online reference site FCz were (re)calculated. After applying a 0.5 Hz high‐pass filter and 30 Hz low‐pass filter with a slope of 12 dB/Oct each (see Luck 2014), blinks and vertical eye movements were removed via an independent component analysis (ICA) procedure: After running the ICA (Infomax algorithm based on the whole data), one component with a frontocentral distribution that showed frontally pronounced positive peaks coinciding with blinks and movements recorded via the vEOG electrodes was identified and removed before an inverse ICA was conducted. Subsequently, the data were segmented: EEG data from the learning blocks, from which the FRN and N170 were extracted (see below), were divided into 800 ms segments, starting 200 ms before feedback onset. EEG data from the primed recognition task, from which the N400 was extracted (see below), were divided into 1200 ms long segments, starting 200 ms before the onset of the (primed) novel word. In the next step, all segments were baseline corrected (baseline was set as the 200 ms before the respective event), and an automatic artifact rejection was performed for those electrodes entering each particular analysis (see below). Segments were excluded from the later analysis if they showed a voltage step of more than 50 μV/ms, a maximum difference of values larger than 100 μV, or amplitudes lower than −100 μV or higher than 100 μV at one or more of the measured electrode sites. On average, 3.0% of all segments were removed per participant across all segmented events (positive feedback, negative feedback, associated target, associated distractor, not associated target, not associated distractor). For detailed information on removed segments, see Section S1. For the EEG data from the learning phase, separate averages were created for positive and negative feedback to later extract peak latencies (see below).

### 1.7 Analysis and ERP Amplitude Extraction

As described in the introduction, the first aim of the study was to investigate if the FRN and/or the N170 in the feedback‐locked ERP during learning can predict behavioral free recall and/or recognition performance. The second and main aim of the study was twofold, and was to investigate (a) if learning of novel‐word‐novel‐object associations in a feedback‐learning task transfers to an N400 priming effect indicative of the semantic association strength between novel object and novel word; (b) if the feedback‐locked FRN and/or N170 during learning can predict the N400 during primed recognition. Although the N400 during priming thus served as dependent variable, the feedback‐locked FRN and N170 amplitudes were used as predictors for three different dependent variables. Before the main analyses targeting our hypotheses were conducted, control analyses on the FRN and N170 served to determine whether we could replicate previously reported result patterns for these components during feedback learning.

Further processing steps were conducted using MATLAB (version 2017b; Mathworks, Natick, Massachusetts, USA). We pooled the electrodes Fz, FCz, and Cz for later FRN extraction, and electrodes P7 and P8 for the N170. This pooling procedure for the ERP components serving as predictors yielded more parsimonious models. For both ERP components, we extracted single‐trial amplitudes during the learning phase based on the peak‐to‐peak approach (see below for details and Albrecht and Bellebaum 2021). In a first step, the latency of the negative peak in a time window between 200 and 400 ms after feedback was determined for the FRN in the averages at the pooled signal, separately for positive and negative feedback and each participant. Subsequently, the latency of the preceding positive peak, also separately for positive and negative feedback and each participant, was determined in the averages in a time window between 100 ms and the negative peak latency for later amplitude calculation on the single‐trial level based on the peak‐to‐peak approach. For the N170, the procedure was the same, with a time window between 110 and 260 ms for the negative peak, and between 30 ms and the latency of the negative peak for the preceding positive peak at the pooled signal of P7 and P8. In a second step, the amplitude values at the previously determined latencies of the negative and preceding positive peaks were extracted from the single‐trial data by calculating the mean in an area from 10 ms before to 10 ms after the peak latencies corresponding to the feedback valence condition of the single trial (positive vs. negative feedback). In a third step, amplitude values based on the peak‐to‐peak approach for single trials were calculated by subtracting the amplitude value corresponding to the latency of the positive peak in this trial from the amplitude value corresponding to the latency of the negative peak.

In the analysis of the N400 priming effect, we also aimed to determine if the N400 is most pronounced over frontal or parietal sites and if it shows a lateralization in its topography. We thus analyzed the N400 at the 11 fronto‐centro‐parietal electrode sites F3, Fz, F4, FC1, FCz, FC2, CP1, CP2, P3, Pz, and P4. According to standard procedures for the N400 (Kutas and Federmeier 2011), we extracted the N400 amplitude as an area mean at the single‐trial level. Visual inspection of grand average waveforms showed that the N400 was most pronounced between 270 and 470 ms. Thus, we used the area mean in this time frame for each single‐trial segment, separately for all electrode sites, as a measure for the N400 in our analyses. Please note that we did not pool over electrode sites for the N400, because, as a dependent variable, individual values per site carry valuable information and topographical variations of N400 priming effects have been previously reported in the recognition of novel words (Leynes and Upadhyay 2022). After an N400 priming effect analysis including central (Fz, FCz, and Pz), left hemispheric (F3, FC1, CP1, and P3), and right hemispheric (F4, FC2, CP2, and P4) electrodes at anterior (Fz, F3, F4, FCz, FC1, and FC2) and posterior sites (CP1, CP2, Pz, P3, and P4) confirmed that the N400 priming effect was strongest at midline frontocentral sites (see below), we restricted the following FRN‐N400 and N170‐N400 analysis to electrode sites Fz and FCz.

### 1.8 Statistical Data Analysis

#### 1.8.1 Behavioral Analyses

##### 1.8.1.1 Learning Blocks

Accuracy was calculated as the mean percentage of trials participants chose the associated target, separately for each learning block and Feedback Timing condition. For the statistical analysis of accuracy, we fitted a GLME model including the fixed effects Feedback Timing (immediate [−0.5], delayed [0.5]) and Block (1–5, scaled and mean‐centred), as well as random intercepts by participant.

Reaction times were measured from the onset of the novel‐object‐novel‐word combination to the participants' response. An LME model was fitted to log‐transformed reaction times, including the fixed effects Feedback Timing, Response Correctness (correct [0.5], wrong [−0.5]; because feedback was deterministic, this factor corresponded to Feedback Valence) and Block (1–5, scaled and mean‐centred), as well as random intercepts by participant and random slopes for Block and for Response Correctness by participant.

##### 1.8.1.2 Free Recall Test

Accuracy was calculated as the mean percentage of trials participants correctly entered the associated target word (allowing for deviations of one character). Accuracy in the free recall test was statistically investigated as part of the FRN‐Recall and N170‐Recall analyses (see below), including effects of Feedback Timing on recall accuracy. Because in the free recall test participants typed their response without a time limit, reaction times were not considered meaningful and were thus not analyzed.

##### 1.8.1.3 Primed Recognition Task

Accuracy was calculated as the percentage of novel objects for which participants correctly accepted the associated target novel word and correctly rejected the three unrelated novel words (i.e., the associated distractor, the not associated target and the not associated distractor words). Accuracy in the primed recognition task was statistically investigated as part of the FRN‐Recognition and N170‐Recognition analyses (see below), including effects of Feedback Timing on recognition accuracy.

Reaction times were measured from the onset of the question to the participants' response. An LME model was fitted to log‐transformed reaction times including the fixed effects Feedback Timing, Response Correctness, and Priming Condition (associated target, associated distractor, not associated target, not associated distractor), as well as random intercepts by participant and random slopes for Response Correctness by participant.

#### 1.8.2 ERP Control Analyses

To validate the informative value of the measured FRN and N170 amplitudes, we set up control analyses to examine their modulation by feedback timing and feedback valence based on the extant literature (e.g., Arbel et al. 2017; Höltje and Mecklinger 2020; Peterburs et al. 2016). For these FRN and N170 control analyses, we set up a linear mixed effects (LME) model and determined Feedback Timing (immediate [−0.5], delayed [0.5]) and Feedback Valence (negative [−0.5], positive [0.5]) as fixed effects. We also allowed random intercepts by subject and random slopes for Feedback Valence by subject, resulting in the models:

(1) $$\begin{array}{c} \mathrm{FRN}\sim \text{Feedback Timing}*\text{Feedback Valence}\\ \;+\left(1+\text{Feedback Valence}\;|\;\text{Subject}\right) \end{array}$$

(2) $$\begin{array}{c} N170\sim \text{Feedback Timing}*\text{Feedback Valence}\\ \;+\left(1+\text{Feedback Valence}\;|\;\text{Subject}\right) \end{array}$$

To remove outliers from each dependent variable in each model of the control analyses, we set up the respective model as described above and subsequently removed data points with residual values that deviated more than 2 standard deviations from the mean of all residual values before recalculating the model. In the subsequent main analyses, we used the outlier‐corrected datasets for the analyses including the respective component as a predictor (FRN‐outlier removed for subsequent analyses involving the FRN as effect, N170‐outlier removed for subsequent analyses involving the N170 as effect). Even though FRN and N170 amplitudes were used as predictors in the main analysis models, they represent measured brain activity that is susceptible to noise. However, a residual‐based outlier removal addresses outliers in the dependent variable, not in the predictors. Therefore, we adopted a two‐step procedure: we first removed outliers of the FRN and N170 from the dataset based on the residuals in the control analysis models, and subsequently removed outliers for the dependent variable N400 based on residuals in the main analysis models.

#### 1.8.3 ERP Main Analyses

##### 1.8.3.1 Prediction of Behavioral Free Recall Performance

Because the results of the control analyses showed comparable patterns for FRN and N170 (see below), we assumed that the two variables were not independent from each other. We thus decided to calculate separate models including either FRN or N170 amplitude as predictors for each of the dependent variables. We therefore created two generalized linear mixed effect (GLME) models to analyze if the feedback‐locked FRN and/or the N170 during learning predicted later free recall performance, with free recall performance as a binary dependent variable, coded as 0 (incorrect) and 1 (correct). For a given novel object, free recall was considered correct if not more than one letter differed from the correct novel word (substitution, addition, or reduction). For each trial of the learning phase, we thus determined whether participants could later recall the associated word for the object presented in this trial or not. In separate analyses, we then tested whether the FRN or the N170 in response to the feedback that participants received for their choice of word for a given object could predict later recall performance. We thus determined one model that included the continuous predictor FRN, which was scaled to vary between 0 and 1 and then mean‐centered (FRN‐Recall model), and one model that included the continuous predictor N170, which was scaled and mean‐centered in the same way (N170‐Recall model). In both models, we included feedback delay as a between‐subject variable, coded as −0.5 (immediate) and 0.5 (delayed). We also included feedback valence (positive vs. negative) as a nested factor (nested in FRN and N170, respectively) that further modulated FRN and N170 effects, coded as −0.5 (negative) and 0.5 (positive).

We allowed random intercepts and slopes per participant. Ideally, all within‐subject main and interaction effects would be included as random effects in the model (according to best practice, Meteyard and Davies 2020). Thus, random intercepts and slopes per participant were included using the highest complex model possible, as long as their inclusion did not lead to over‐ or underfitting. This was determined using the function buildmer (version 2.11). Subsequently, we tested for the most parsimonious model by stepwise elimination, checking for model fit changes, also using the function buildmer (version 2.11). As a result, the FRN‐Recall model was built as follows:

(3) $$\begin{array}{c} \text{Free Recall Accuracy}\sim \mathrm{FRN}*\text{Feedback Timing}\\ \;+\text{Feedback Valence}:\mathrm{FRN}*\text{Feedback Timing}\\ \;+\left(1+\mathrm{FRN}+\text{Feedback Valence}:\mathrm{FRN}|\text{Subject}\right) \end{array}$$

and the N170‐Recall model as this:

(4) $$\begin{array}{c} \text{Free Recall Accuracy}\sim N170*\text{Feedback Timing}\\ \;+\text{Feedback Valence}:N170*\text{Feedback Timing}\\ \;+\left(1+N170+\text{Feedback Valence}:N170|\text{Subject}\right) \end{array}$$

##### 1.8.3.2 Prediction of Behavioral Recognition Performance

To test whether recognition performance can be predicted from feedback‐locked ERP components during learning, we again determined a GLME model with recognition performance as a binary dependent variable (coded as 0 for incorrect, 1 for correct). For each novel object, recognition was considered correct when participants (i) correctly associated the novel object with the associated target novel word, and (ii) correctly rejected the associated distractor, the not associated target, and the not associated distractor novel word. In turn, recognition was considered incorrect if one of these criteria was not met. Again, we determined separate models including either the FRN [FRN‐Recognition model] or N170 [N170‐Recognition model]. We included the same between‐ and within‐subject effects as for the free recall performance models. As in the previous models, we allowed random intercepts and slopes per participant, and included all within‐subject effects possible using the buildmer function (version 2.11), resulting in the models:

(5) $$\begin{array}{c} \text{Recognition Accuracy}\sim \mathrm{FRN}*\text{Feedback Timing}\\ \;+\text{Feedback Valence}:\mathrm{FRN}*\text{Feedback Timing}\\ \;+\left(1\;|\text{Subject}\right) \end{array}$$

(6) $$\begin{array}{c} \text{Recognition Accuracy}\sim N170*\text{Feedback Timing}\\ \;+\text{Feedback Valence}:N170*\text{Feedback Timing}\\ \;+\left(1+N170+\text{Feedback Valence}:N170|\text{Subject}\right) \end{array}$$

##### 1.8.3.3 N400 Priming Effect

For the N400 analysis on the priming effect, we set up an LME model with the N400 amplitude as dependent variable and Priming Condition (associated target, associated distractor, not associated target, not associated distractor) as within‐subject factor (we used a simple coding contrast matrix and the associated target was set as baseline), as well as the within‐subject factor Recognition (recognized [0.5], not recognized [−0.5], defined as for the dependent variable in the Recognition models). We also included the three‐level within‐subject factor Laterality, with central (electrodes Fz, FCz, and Pz) as baseline, against which left hemisphere (F3, FC1, CP1, and P3) and right hemisphere (F4, FC2, CP2, and P4) were contrasted, and the two‐level within‐subject factor Frontality, with the levels frontal (0.5, electrodes Fz, F3, F4, FCz, FC1, and FC2) and parietal (−0.5, electrodes CP1, CP2, Pz, P3, and P4). We allowed random intercepts per electrode and random intercepts and slopes per participant. Random effects were included based on the previously described procedure, resulting in the model:

(7) $$\begin{array}{c} N400\sim \text{Priming Condition}*\text{Laterality}*\text{Frontality}*\text{Recognition}\\ \;+\left(1+\text{Laterality}\;|\;\text{Subject}\right)+\left(1\;|\;\text{Electrode}\right) \end{array}$$

##### 1.8.3.4 Prediction of Association Strength Measured by N400 Amplitude

To test whether the association strength between novel objects and novel words as reflected by N400 priming effect can be predicted from feedback‐locked ERP components during learning, we determined two GLME models with N400 amplitude as dependent variable. As the priming effect was most pronounced at frontocentral sites (see Section S2 and Table S3.11), we included only frontocentral electrodes (Fz, FCz) in the main analyses on the N400 to reduce model complexity. Concretely, we tested whether the FRN or N170 in response to feedback which the participants received for their choice of a novel word for a given novel object during learning predicted the N400 for associated or non‐associated words for the same object during the primed recognition test. In the two GLME models including the N400 at frontocentral electrodes as dependent variable, we thus modeled either the continuous within‐subject factor FRN [FRN‐N400 model] or N170 [N170‐N400 model] (scaled and mean‐centered), as well as the factors Feedback Delay (coded as above) and Feedback Valence (nested in FRN or N170, respectively, and coded as described above). As the control conditions, that is, unrelated priming conditions (associated distractor, not associated target, not associated distractor) did not differ in the N400 priming analysis at frontocentral electrode sites (see below and Table S3.11), we combined them into the level “unrelated words” in the FRN‐N400 model and N170‐N400 model to further reduce complexity: we thus included the factor Priming Condition with two levels (associated target [0.5], unrelated words [−0.5]). Because the FRN‐Recognition and N170‐Recognition models aimed to predict recognition accuracy, we refrained from adding the factor Recognition to the FRN‐N400 or N170‐N400 model, as the factors would not be independent, and adding Recognition as a predictor would bear the risk of decreasing variability of the FRN and N170 as predictors. As described above, we set up the models, that is, already including outlier‐adjusted FRN or N170 amplitudes, respectively, and subsequently removed residual values that deviated more than 2 standard deviations from the mean of all residual values before recalculating the model. For the FRN as predictor, we used the model:

(8) $$\begin{array}{c} N400\;\text{Amplitude}\sim \text{Feedback Timing}*\text{Priming Condition}*\mathrm{FRN}\\ \;+\text{Feedback Valence}:\mathrm{FRN}*\text{Feedback Timing}*\text{Priming Condition}\\ \;+\left(1+\text{Priming Condition}*\mathrm{FRN}+\mathrm{FRN}:\text{Feedback Valence}\;|\;\text{Subject}\right) \end{array}$$

And for the N170 as predictor, we used the model:

(9) $$\begin{array}{c} N400\;\text{Amplitude}\sim \text{Feedback Timing}*\text{Priming Condition}*N170\\ \;+\text{Feedback Valence}:N170*\text{Feedback Timing}*\text{Priming Condition}\\ \;+\left(1+\text{Priming Condition}*N170+N170:\text{Feedback Valence}\;|\;\text{Subject}\right) \end{array}$$

## 2 Results

For better readability, we only report significant effects (based on an *α* = 0.05) in the following text. Full inferential statistics, including *t*‐/*z*‐values, *β*‐values, confidence intervals of *β*‐values, as well as the mean number of data points per condition included in the respective analysis for all models can be found in Sections S1 to S3 (see Tables S3.1–S3.14 for detailed information for all models).

### 2.1 Behavioral Results

Participants' accuracy significantly increased across the learning blocks, *z* = 35.13, *p* < 0.001, *β* = 1.77, from an average of 50.05% (SD = 1.17%) in the first block to 83.62% (SD = 13.67%, chance level = 50%) in the last block, with a stronger increase for immediate than delayed feedback, resulting in a significant interaction, *z* = −6.09, *p* < 0.001 (for single‐subject accuracy results, please see Section S1). In the free recall test, participants on average responded correctly in 44.84% of trials (SD = 26.78%), and in the primed recognition test correctly recognized 52.58% of novel‐object‐novel‐word associations (SD = 26.18%, chance level to respond correctly in all four cases was 6.25%). Inferential statistics on free recall and recognition accuracy are reported as part of the FRN‐Recall, N170‐Recall, FRN‐Recognition and N170‐Recognition models (see below), and these analyses did not reveal effects of Feedback Timing on recall or recognition performance. Detailed descriptive and inferential results for accuracy (in the learning blocks, the free recall test and primed recognition task) and reaction times (in the feedback learning blocks and the primed recognition task) are reported in Section S1.

### 2.2 ERP Control Analyses

**FIGURE 2:**
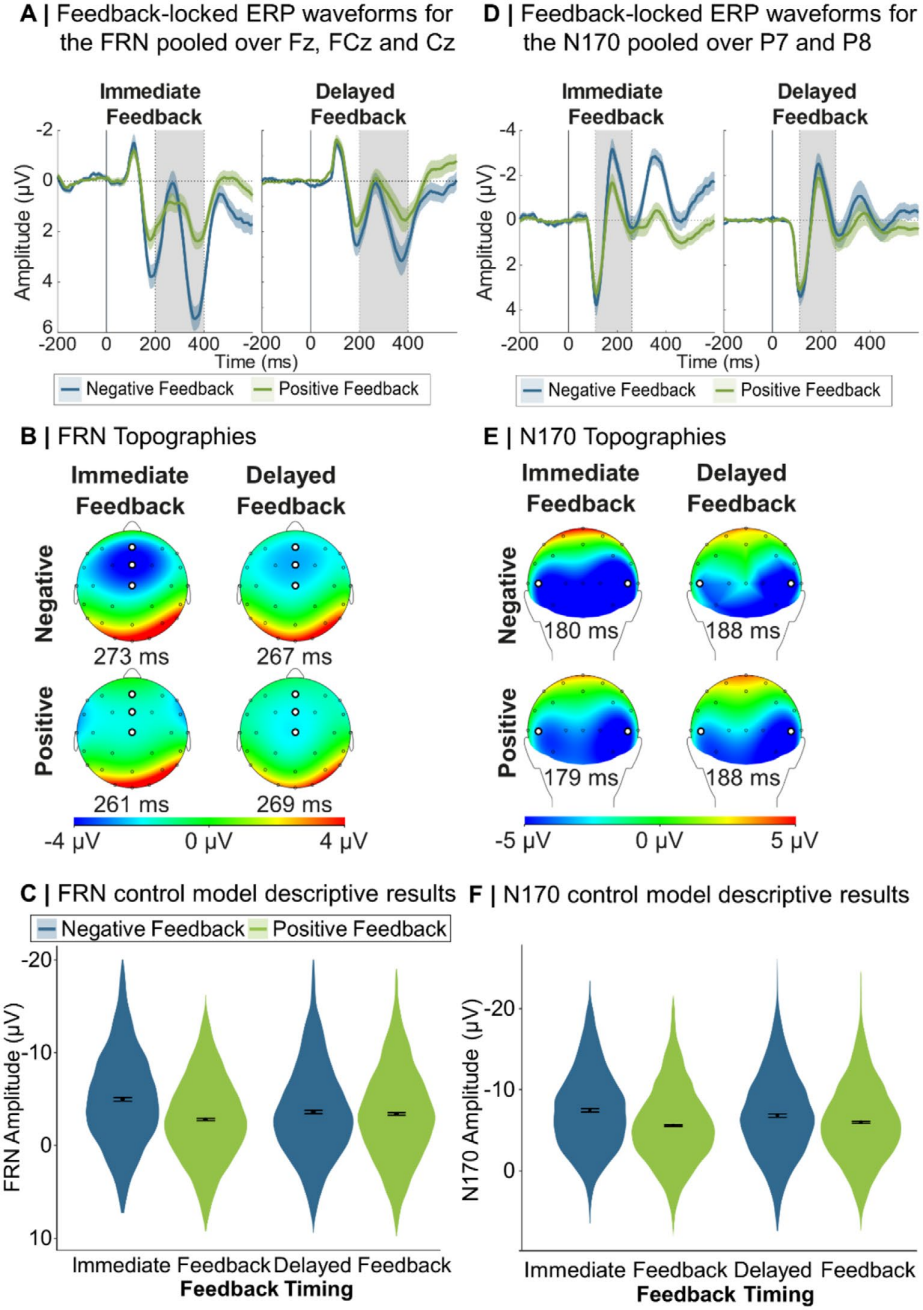
Feedback‐locked grand average ERP waveforms, topographies and control model predictions for the FRN and N170 peak‐to‐peak extraction. Error margins around the grand averages (*n* = 33 in both Feedback Delay conditions) represent ±1 standard error. Gray areas represent the time window in which the maximum negative peaks per condition and participant were searched. Error margins in subplots C and F represent 95% confidence intervals. FRN, feedback‐related negativity.

Figure 2 displays grand averages and topographies of the FRN and N170 for immediate and delayed positive and negative feedback during learning as well as model estimates of the control analysis models.

#### 2.2.1 FRN

For the FRN there was a significant main effect of Feedback Valence, *F*(1,63.51) = 42.07, *p* < 0.001, *β* = 1.30, with higher amplitudes for negative than positive feedback. This was further explained by a Feedback Timing by Feedback Valence interaction (see Figure 2C). Only for immediate feedback, negative feedback resulted in higher FRN amplitudes than positive feedback, *F*(1,64.27) = 54.66, *p* < 0.001, *β* = 2.10 (*p* = 0.083 for delayed feedback).

#### 2.2.2 N170

For the N170 we also found a significant main effect of Feedback Valence, *F*(1,65.09) = 98.81, *p* < 0.001, *β* = 1.55. N170 amplitudes were higher for negative than positive feedback. As for the FRN, we found a Feedback Timing by Feedback Valence interaction, *F*(1,65.09) = 7.11, *p* = 0.010. For both delayed feedback, *F*(1,64.59) = 26.55, *p* < 0.001, *β* = 1.13, and immediate feedback, *F*(1,65.59) = 79.17, *p* < 0.001, *β* = 1.97, amplitudes were higher for negative than positive feedback, while the effect was more pronounced for immediate feedback (see Figure 2F).

### 2.3 ERP Main Analyses

Grand average ERPs showing the FRN and N170 separately for Feedback Timing, Feedback Valence and Recall/Recognition are presented in Figure S3.1, Section S3.

#### 2.3.1 Prediction of Recall Performance

##### 2.3.1.1 FRN‐Recall

**FIGURE 3:**
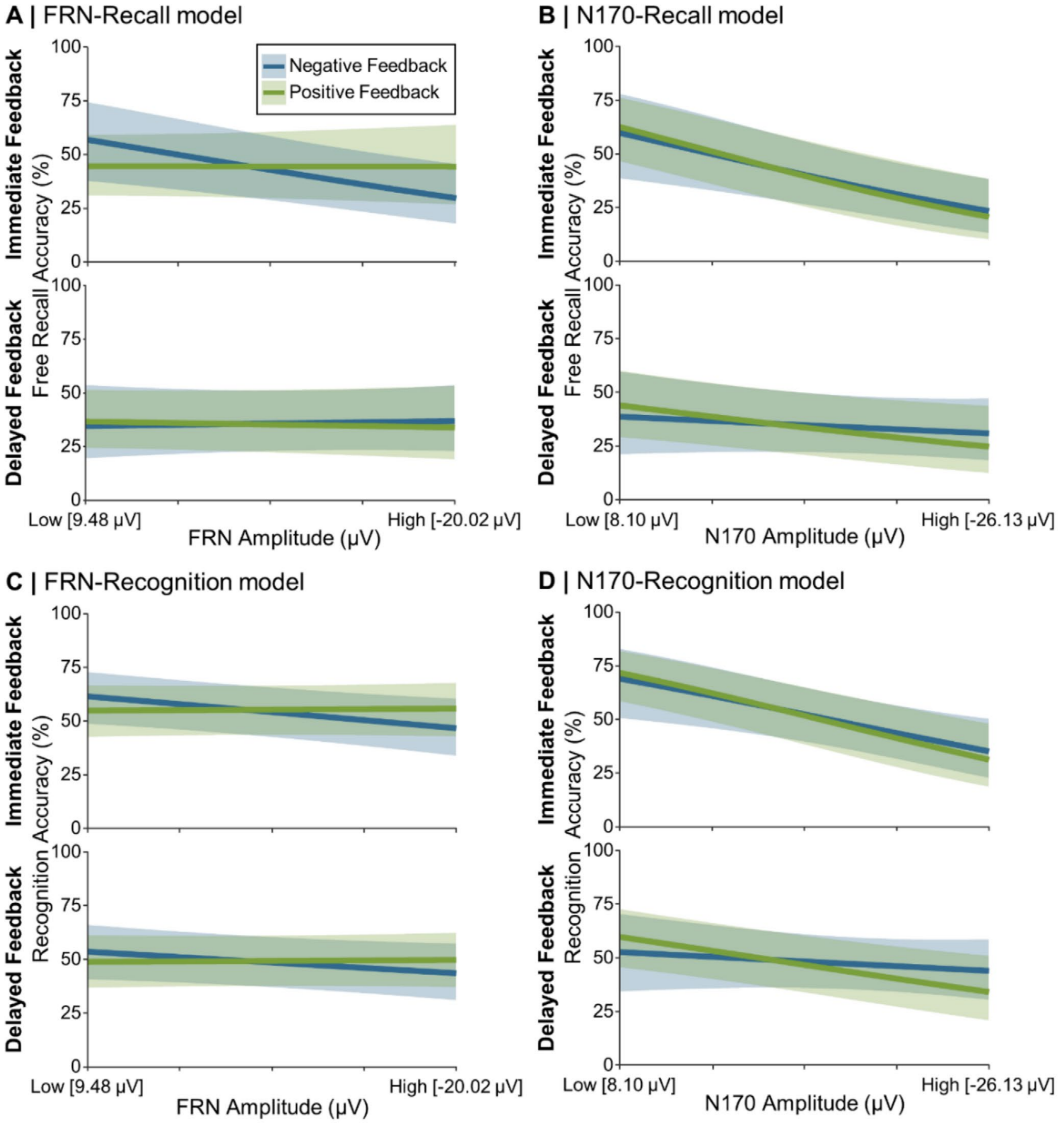
Model estimates for models in which (A) the FRN and (B) the N170 predicted recall, and for the models in which (C) the FRN and (D) the N170 predicted recognition. The blue and green lines depict the GLME predictions based on fitted *β* weights and intercepts. Error margins represent 95% confidence intervals of the model predictions. Amplitude of the FRN and N170 as predictors on the *x*‐axes are depicted from least to most negative (i.e., low to high amplitude). FRN, feedback‐related negativity.

No significant effects emerged for the analysis in which the FRN was used as a predictor for free recall performance. Model estimates for the FRN‐Recall are displayed in Figure 3A; an estimation of the Bayes Factor for the null effect is reported in Section S4.

##### 2.3.1.2 N170‐Recall

For the analysis including the N170 as predictor, there was a significant main effect of N170 Amplitude on free recall performance, *z* = 6.06, *p* < 0.001, *β* = 1.16. The smaller the N170 Amplitude, the better was the free recall accuracy. The N170 Amplitude further interacted significantly with Feedback Timing, *z* = −3.09, *p* = 0.002. The significant N170 Amplitude effect on free recall performance was more pronounced for immediate, *z* = 6.39, *p* < 0.001, *β =* 1.71 than delayed feedback, *z* = 2.35, *p* = 0.019, *β =* 0.60. Model estimates for the N170‐Recall model are displayed in Figure 3B.

#### 2.3.2 Prediction of Recognition Performance

##### 2.3.2.1 FRN‐Recognition

We found a significant main effect of FRN Amplitude on recognition performance, *z* = 2.05, *p* = 0.041, *β* = 0.26. The smaller the FRN, the better the recognition performance. The FRN Amplitude further interacted significantly with Feedback Valence, *z* = −2.49, *p* = 0.013. The described FRN Amplitude effect was only significant for negative feedback, *z* = 2.80, *p* = 0.005, *β* = 0.55 and not for positive feedback (*p* = 0.801). Model estimates for the FRN‐Recognition model are displayed in Figure 3C.

##### 2.3.2.2 N170‐Recognition

There was a significant main effect of N170 Amplitude on recognition performance, *z* = 5.84, *p* < 0.001, *β* = 1.13. The smaller the N170 Amplitude, the better the recognition performance. The N170 Amplitude further interacted significantly with Feedback Timing, *z* = −2.29, *p* = 0.022. The described N170 Amplitude effect was more pronounced for immediate feedback, *z* = 5.62, *p* < 0.001, *β* = 1.55, than for delayed feedback, *z* = 2.69, *p* = 0.007, *β* = 0.70. Model estimates for the FRN‐Recognition model are displayed in Figure 3D.

#### 2.3.3 N400 Priming Effect Analysis

**FIGURE 4:**
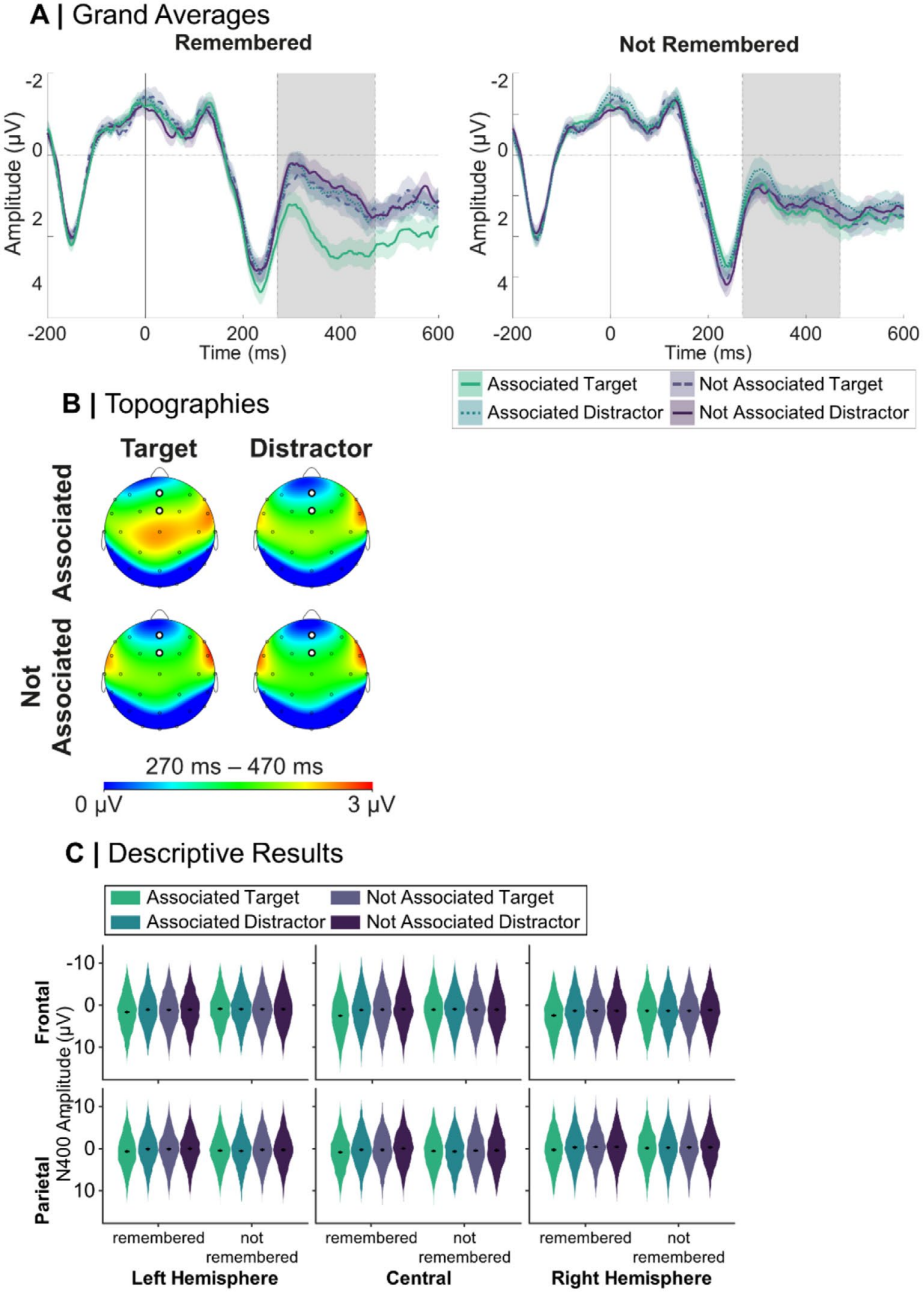
Grand averages, topographies and model predictions of N400 by priming condition. In subplot A, error margins around the grand averages (*n* = 66) represent ±1 standard error, and the gray area represents the time window in which the mean amplitude was calculated as a measure for the N400. In subplot C, error margins represent 95% confidence intervals.

For a display of the grand averages and topographies of N400 amplitudes of the primed recognition task, pooled over the electrodes Fz and FCz for each Priming Condition, as well as model estimates for the N400 priming model, see Figure 4. Concerning the N400 in the primed recognition task, there was a significant main effect of Priming Condition, *F*(3,721144.09) = 610.28, *p* < 0.001. Planned contrasts revealed that N400 amplitudes were significantly smaller in the associated target condition compared to the associated distractor, *t* = −30.13, *p* < 0.001, *β* = −0.40, the not associated target, *t* = −33.39, *p* < 0.001, *β* = −0.44, and the not associated distractor condition, *t* = −38.99, *p* < 0.001, *β* = −0.52. Planned contrasts further revealed smaller N400 amplitudes for the associated distractor than the not associated distractor (*p* < 0.001, *β* = 0.11), significantly smaller amplitudes for the associated distractor than the not associated target (*p* = 0.001, *β* = 0.04), and significantly smaller amplitudes for the not associated target than the not associated distractor (*p* < 0.001, *β* = −0.07). We also found a significant main effect of Recognition, *F*(1,720062.48) = 56.65, *p* < 0.001, *β* = 0.08, with smaller N400 amplitudes for remembered compared to not remembered novel objects. Furthermore, we found a significant interaction between Priming Condition and Recognition, *F*(3,721143.93) = 458.52, *p* < 0.001. For both remembered, *F*(3,721144.17) = 1120.41, *p* < 0.001, and not remembered novel objects, *F*(3,721143.86) = 12.53, *p* < 0.001, significant effects of Priming Condition emerged. For remembered novel objects, N400 amplitudes were significantly smaller for the associated target condition compared to all other priming conditions (associated distractor, *p* < 0.001, *β* = −0.80, not associated target, *p* < 0.001, *β* = −0.84, not associated distractor, *p* < 0.001, *β* = −0.93). For not remembered novel objects, significantly smaller N400 amplitudes emerged for the associated target condition compared to the not associated target condition (*p* = 0.008, *β* = −0.05) and the not associated distractor condition (*p* < 0.001, *β* = −0.10), but not compared to the associated distractor condition (*p* = 0.897).

Finally, we found a significant four‐way interaction between Priming Condition, Recognition, Laterality, and Frontality, *F*(6,721143.81) = 10.20, *p* < 0.001. The aforementioned interaction between Priming Condition and Recognition was present at all possible sites (frontal left, frontocentral, frontal right, parietal left, parietal central, parietal right, all *p* < 0.001), but most pronounced at frontocentral sites, *F*(3,721160.77) = 297.33, *p* < 0.001. For the completely resolved interaction, see Table S3.11, Section S3.

#### 2.3.4 Prediction of Association Strength Measured by N400 Amplitude

##### 2.3.4.1 FRN‐N400

**FIGURE 5:**
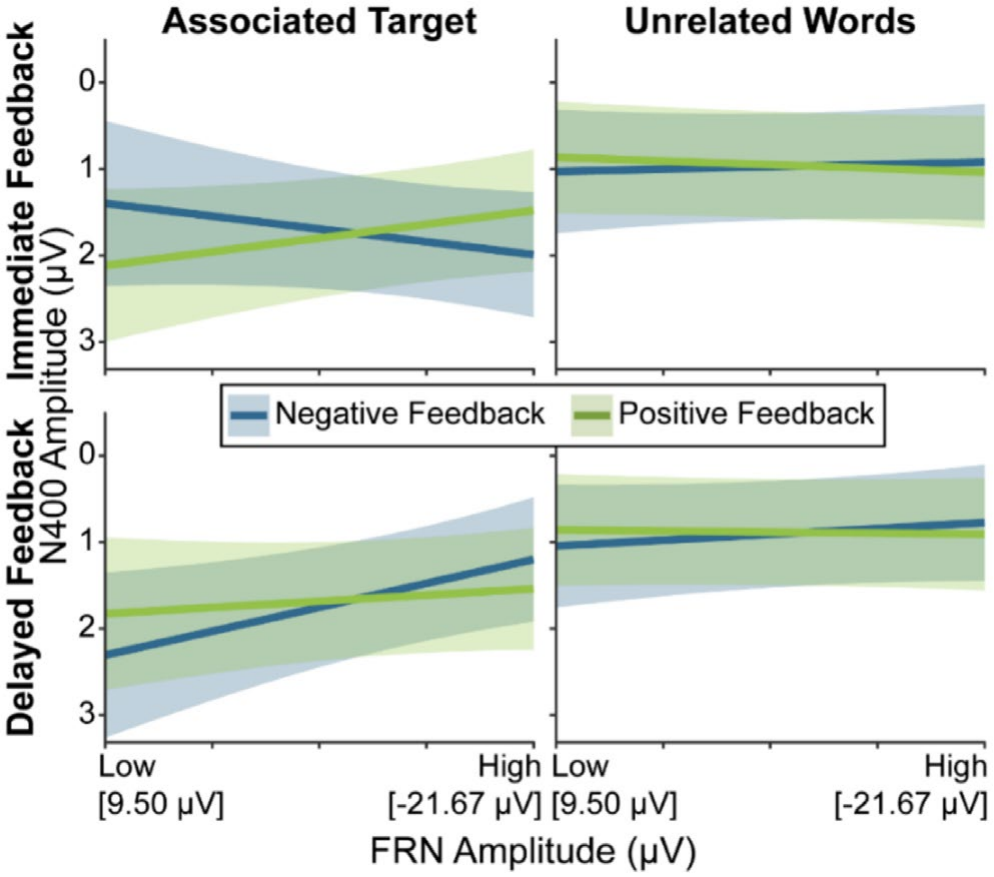
Model estimates for the FRN‐N400 model. The blue and green lines depict the GLME predictions based on fitted *β* weights and intercepts. Error margins represent 95% confidence intervals of the model predictions. Amplitude of the FRN as a predictor on the *x*‐axis is depicted from least to most negative (i.e., low to high amplitude). FRN, feedback‐related negativity. For a scatter plot of this figure that shows individual datapoints as well as means and confidence intervals of the raw data, please refer to Figure S3.2, Section S3.

Model estimates for this analysis are depicted in Figure 5. In the analysis in which the FRN was used to predict N400 amplitudes, there was a significant main effect of Priming Condition on the N400, *F*(1,64.21) = 33.66, *p* < 0.001, *β* = 0.78. As reported in the priming model, participants showed significantly reduced N400 amplitudes for the associated target condition compared to the unrelated words condition. A significant three‐way interaction between Feedback Timing, FRN, and Feedback Valence, *F*(1,80.03) = 4.27, *p* = 0.042, could be further explained by a four‐way interaction between Priming Condition, Feedback Timing, FRN, and Feedback Valence, *F*(1,46569.74) = 6.93, *p* = 0.008. A stepwise resolution of this interaction, by keeping Priming Condition constant on each level, revealed a significant interaction between Feedback Timing, FRN Amplitude, and Feedback Valence only for the associated target condition, *F*(1,363.54) = 7.66, *p* = 0.006, not for the unrelated words condition (*p* = 0.935). Further resolving for Feedback Valence revealed a Feedback Timing by FRN Amplitude interaction for negative, *F*(1,106.16) = 5.82, *p* = 0.018, but not for positive feedback (*p* = 0.553). Finally, an FRN Amplitude effect emerged only for delayed negative feedback in the associated target condition, *F*(1,104.24) = 5.04, *p* = 0.027, *β* = −1.11, with less pronounced N400 amplitudes for smaller FRN amplitude values (*p* = 0.241 for immediate feedback). Exploratory analyses separately for all conditions (Priming Condition × Feedback Timing × Feedback Valence) revealed that in none of the other conditions a significant FRN amplitude effect was found (*p* ≥ 0.128).

##### 2.3.4.2 N170‐N400

**FIGURE 6:**
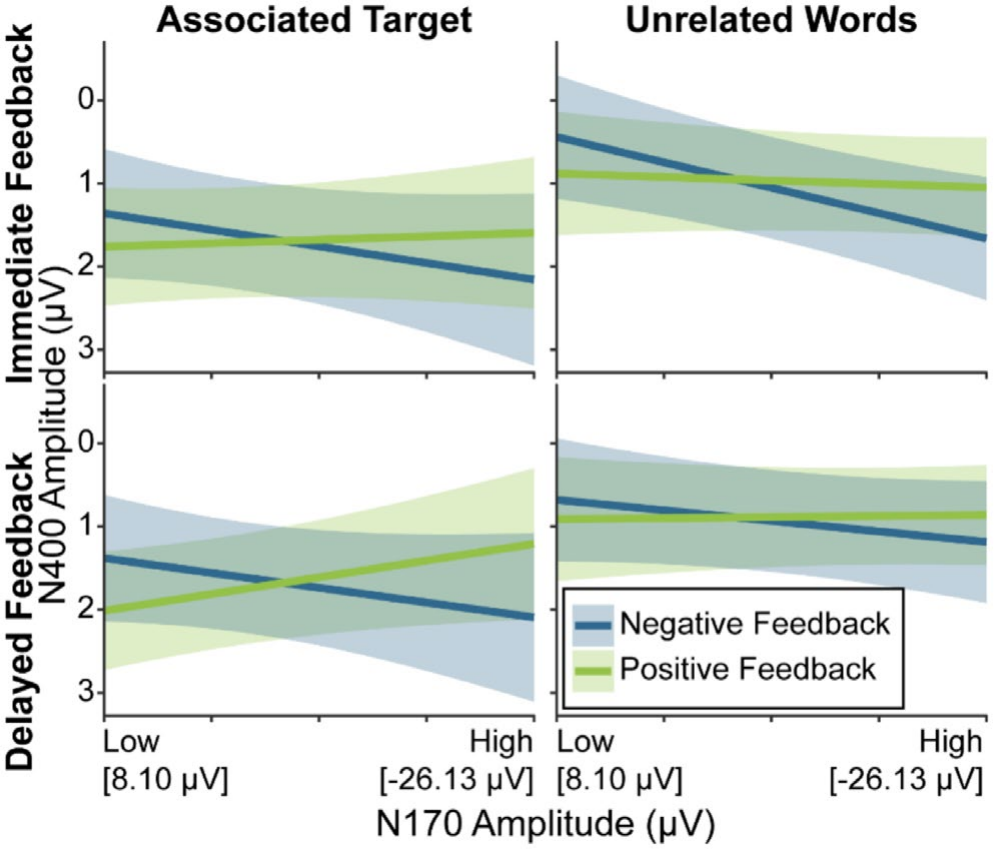
Model estimates for the N170‐N400 model. The blue and green lines depict the GLME predictions based on fitted *β* weights and intercepts. Error margins represent 95% confidence intervals of the model predictions. Amplitude of the N170 as predictor on the *x*‐axis is depicted from least to most negative (i.e., low to high amplitude). For a scatter plot of this figure that shows individual datapoints as well as means and confidence intervals of the raw data, please refer to Figure S3.3, Section S3.

Figure 6 displays model estimates for this analysis. For the analysis in which the N170 was used to predict the N400, as in the FRN‐N400 model, we found again a main effect of Priming Condition, *F*(1,63.91) = 31.31, *p* < 0.001, *β* = 0.76 (see above). We further found a significant interaction between N170 Amplitude and Feedback Valence. Manual simple slope analyses showed that an N170 Amplitude effect emerged only for negative feedback, *F*(1,72.58) = 8.02, *p* = 0.006, *β* = −0.81. Participants showed reduced N400 amplitudes for higher N170 amplitudes.

## 3 Discussion

In the current study, we first aimed to link feedback‐locked ERP components during the feedback‐based learning of novel‐object‐novel‐word associations, that is, the FRN and N170, with subsequent memory performance (free recall and recognition), as in our previous related study (Albrecht et al. 2023). In order to address our main research question, we examined if the strength of the associations acquired through feedback‐based learning yields N400 priming effects and, more crucially, if the feedback‐locked FRN and N170 during learning predict the strength of these N400 priming effects. Participants learned novel‐object‐novel‐word associations with immediate or delayed feedback and learning performance was then assessed in a free recall and a primed recognition task. Participants' accuracy steadily increased throughout the learning blocks and showed accuracy rates well above chance level in the free recall test and primed recognition task. While accuracy in the learning blocks increased slightly more for immediate than delayed feedback, accuracy in the free recall test and primed recognition task did not differ between participants who learned with immediate or delayed feedback. Together, these findings indicate robust acquisition and retention of the novel associations.

As expected, feedback‐based learning of novel‐object‐novel‐word associations led to N400 priming effects. Words preceded by associated objects elicited smaller N400 amplitudes compared to words preceded by not‐associated objects. Partially in line with our hypotheses, smaller FRN amplitudes in response to negative feedback during learning predicted better recognition performance as well as a stronger association reflected by the N400 priming effect. Also partially as expected, lower N170 amplitudes predicted recall performance as well as recognition performance, irrespective of valence. Further, higher N170 amplitudes during learning predicted facilitated semantic processing reflected in N400 reductions; however, irrespective of established associations.

First of all, our control analyses suggest that the FRN and N170 are indeed reliable markers of feedback‐based learning as they showed the typical pattern with higher FRN amplitudes after negative than positive feedback only for immediate and not delayed feedback (see Albrecht et al. 2023; Höltje and Mecklinger 2020; Peterburs et al. 2016; Röhlinger, Albrecht, and Bellebaum 2025; Röhlinger, Albrecht, Ghio, and Bellebaum 2025; Weinberg et al. 2012), and a similar pattern for the N170 (replicating our previous study, Albrecht et al. 2023, but in contrast to Arbel et al. 2017; Höltje and Mecklinger 2020; Kim and Arbel 2019). This clearly speaks to the informative value of the two ERP predictors, which lays the foundation for the following interpretations regarding the FRN and N170 as predictors of learning success.

### 3.1 Prediction of Free Recall Performance

As expected, we found no evidence that the FRN predicted later free recall performance, replicating results from our previous study (Albrecht et al. 2023), which are further in line with Staresina and Davachi (2006), who found no effect of striatum or prefrontal cortex activity on free recall performance (see also Flowers et al. 1984). To substantiate the null‐finding, we conducted model comparisons of the FRN‐Recall model reported above and a model excluding the predictor FRN (and valence) and estimated a Bayes Factor from the difference of the Bayesian Information Criteria between the two models (see Section S4). The obtained BF_10_ < 0.01 suggests extreme evidence against any main or interaction effect of FRN on recall performance (Lee and Wagenmakers 2014).

Further in line with our hypotheses, lower N170 amplitudes during learning predicted a better free recall performance (see also Albrecht et al. 2023). Opposed to our expectations, however, we found this effect to be stronger for immediate than delayed feedback. Feedback processing has been reported to shift from striatal to MTL structures when feedback is delayed (Foerde et al. 2013; Foerde and Shohamy 2011a). Accordingly, previous studies found the N170, which has been linked to MTL activity, to be larger for delayed compared to immediate feedback (Arbel et al. 2017; Höltje and Mecklinger 2020; Kim and Arbel 2019). In the current study, as well as our previous study (Albrecht et al. 2023), however, N170 amplitudes were more pronounced for immediate feedback, and the stronger N170 effect on free recall performance for immediate feedback in the current study fits into that pattern. Our paradigm was taken from the study by Arbel et al. (2017), with the crucial difference that our participants expected the newly introduced free recall test. Test expectations have been shown to affect especially free recall performance (Balota and Neely 1980; Neely and Balota 1981) and even more so when participants perform the tests repeatedly, as in our case (Rivers and Dunlosky 2021; Weinstein et al. 2014). Speculatively, the expectation of the free recall test, together with the deterministic (as Arbel et al. 2017) rather than probabilistic feedback (compare, e.g., Höltje and Mecklinger 2020), might thus have led to a strategic activation of the MTL during immediate feedback processing, resulting in the observed higher predictive power of the N170 in response to immediate compared to delayed feedback for learning outcome (see also Section S3.2 below). In processing delayed feedback, the MTL may have been involved anyway (Foerde et al. 2013; Foerde and Shohamy 2011a), so that the link between the N170, presumably reflecting MTL activity (Arbel et al. 2017; Höltje and Mecklinger 2020; Kim and Arbel 2019), and learning may have been less pronounced, albeit still significant. This strategic MTL activation may have even been particularly effective for immediate feedback, since participants' accuracy in the learning blocks increased more for immediate than delayed feedback (although no feedback timing effect emerged for the free recall test after learning).

The effect of lower N170 amplitudes predicting better free recall performance was not influenced by feedback valence. A major limitation to the interpretability of valence effects was that, due to the deterministic feedback, there were very few negative feedback events in later blocks (mean accuracy in the final block was about 84%). As a consequence, we were not able to analyze data across blocks or directly model the prediction error. One explanation for this result may be found in the prediction error coding by the N170: Two very recent studies from our group showed prediction error processing in the N170 (Röhlinger, Albrecht, and Bellebaum 2025; Röhlinger, Albrecht, Ghio, and Bellebaum 2025), where expected positive and unexpected negative outcomes led to lower N170 amplitudes relative to unexpected positive and expected negative feedback, respectively. Due to the high learning performance, unexpected negative outcomes were more frequent at the beginning of learning, while expected positive outcomes became more and more frequent at later stages of learning. Speculatively, MTL involvement in both unexpected negative and expected positive feedback might have been specifically important for later free recall performance by first forming declarative memories and then enforcing those memories, respectively.

### 3.2 Prediction of Recognition Performance

In line with our hypotheses, we found effects of both the FRN and the N170 on recognition performance. A smaller FRN in response to negative feedback predicted better recognition performance. Interestingly, this effect was independent of feedback timing, possibly because learning strategies were more strongly influenced by test‐expectancy‐driven learning strategies (see above) than feedback timing. Carefully contextualizing the specificity of this effect for negative valence, previous studies showed that the FRN is smaller for expected than unexpected negative feedback (Hoy et al. 2021; Rawls and Lamm 2021; Röhlinger, Albrecht, and Bellebaum 2025; Röhlinger, Albrecht, Ghio, and Bellebaum 2025). Thus, the reduced FRN in these cases could indicate that negative feedback for the choice of a not‐associated word for a given object was expected, suggesting that learning had taken place. Notably, we found no effect on recognition performance of the FRN in response to positive feedback, where higher FRN amplitudes would indicate more expected positive feedback (see e.g., Burnside et al. 2019; Weber and Bellebaum 2024). This might be due to the fact that positive feedback occurred more often at later learning stages (see above) when association had already been established, and that there was less variability in FRN amplitudes in response to positive feedback.

For the N170, we observed the same pattern for recognition as for free recall performance: the smaller the N170, the better the recognition performance, with stronger effects when feedback was immediate (but again no main effect of feedback timing). Again, the stronger effects for immediate feedback might be due to a focus on declarative learning strategies (see above). This consistent pattern over both memory tasks is a novel finding and suggests that conscious recall processes already triggered by object presentation might have been similar in both tasks, leading to improved performance.

The differential patterns of FRN and N170 amplitudes predicting free recall and recognition performance suggest that the two components represent partially different mechanisms of memory encoding that result in different memory representations. Taken together with the findings that higher connectivity between striatum and hippocampus during encoding leads to better recognition performance (Sadeh et al. 2011), it seems likely that the declarative and procedural memory systems worked in tandem, with the MTL reinforcing existing memories and the striatum signaling the need to adapt associations (see also Röhlinger, Albrecht, Ghio, and Bellebaum 2025). This hypothesis needs to be examined further with a different experimental task in which prediction error modeling is possible.

### 3.3 N400 Priming Effects

As expected, we found a strong N400 priming effect over all electrode sites, with the effect being most pronounced at midline frontocentral sites. Specifically, novel words elicited significantly reduced frontocentral N400 amplitudes when primed by the associated novel object compared to the three unrelated word conditions in which a not‐associated object was presented as prime. This priming effect was especially pronounced for correctly recognized novel‐word‐novel‐object associations. In turn, for associations that were not correctly recognized, both the associated target and associated distractor elicited slightly smaller N400 amplitudes compared to not associated words, possibly reflecting the uncertainty in the acquired associations. This priming effect aligns with previous training studies reporting N400 reductions for successfully acquired associations (Bermúdez‐Margaretto et al. 2019; Borovsky et al. 2012; Dobel et al. 2010; Korochkina et al. 2024), and extends these findings to the specific case of picture‐driven novel word priming after feedback‐based association learning. In our case, the frontocentral rather than classically parietal distribution of the N400 effect might be due to the repeated presentation of the stimuli during learning (Voss and Federmeier 2011). Alternatively, it might suggest a functional distinction with a larger weight put on old/new recognition (i.e., learning induced familiarity), as has been shown in an episodic recognition task (Bridger et al. 2012).

### 3.4 Prediction of Association Strength as Reflected in the N400

Regarding association strength measured via the frontocentral N400 priming effects, a smaller FRN during learning predicted N400 reductions when the associated target word followed the respective object, indicating stronger object‐word associations. In accordance, such priming effects are thought to involve automatic, non‐declarative processes (Grossi 2006; Kiefer 2002). In line with our fronto‐centrally pronounced N400 modulation, the acquired association might have reduced processing effort of the novel target word (Lau et al. 2008) probably through a heightened predictability of the target (Lau et al. 2013). As discussed above, smaller FRN amplitudes in response to specifically negative feedback reflect small prediction errors and thus successful predictions during learning. In this sense the finding is in line with the result for recognition memory: Lower FRN amplitudes, indicating successful prediction of negative feedback, predicted both better recognition as well as stronger object‐word associations. However, the latter effect was specific for delayed feedback. This might be again due to strategic compensation mechanisms as vice versa discussed above for the N170 and immediate feedback: as striatal‐learning is generally favored for immediate feedback (i.e., Foerde and Shohamy 2011a), the link between FRN, representing reward system activity (Becker et al. 2014; Chau et al. 2018; Hauser et al. 2014), and learning might have been less pronounced.

In contrast to the FRN, a higher N170 for negative feedback led to significantly reduced N400 amplitudes. As we described in two recent studies (Röhlinger, Albrecht, and Bellebaum 2025; Röhlinger, Albrecht, Ghio, and Bellebaum 2025), higher N170 amplitudes for negative feedback indicate low prediction errors, and thus expected negative feedback (as assumed for low FRN amplitudes). However, as this prediction of the N400 occurred for the associated target as well as the unrelated words condition, the N170 did not specifically predict the association strength. The N400 has been shown to be reduced for more frequently occurring real words (Allen et al. 2003; Van Petten and Kutas 1990), supposedly reflecting their facilitated processing due to familiarity, and a similar effect might have occurred here. Thus, our results might suggest a more in‐depth encoding, yielding a generally facilitated processing (indicated by reduced N400 amplitudes), for novel words, for which negative feedback processing during learning relied more strongly on MTL involvement (indicated by enhanced N170). The fact that the N170 effect occurred irrespective of timing might again be explained by the above discussed strategy adaptation towards MTL‐based learning as a preparation for the upcoming free recall tests (Balota and Neely 1980; Neely and Balota 1981; Rivers and Dunlosky 2021). The specificity for negative valence of this facilitation effect is in line with findings of larger N170 amplitudes for expected than unexpected negative feedback (Röhlinger, Albrecht, and Bellebaum 2025; Röhlinger, Albrecht, Ghio, and Bellebaum 2025).

An alternative explanation for the missing evidence for condition‐specific N170 effects on the frontal N400 could lie in a possible functional specificity of anterior vs. posterior N400 amplitudes. The frontal N400 has been reported to be specifically involved in familiarity encoding (Curran and Hancock 2007) especially after associative learning (Opitz and Cornell 2006), whereas the N170 might be rather related to more declarative recognition and integration processes, which then might be rather reflected in parietal N400 amplitudes (as in the classical anomalous sentence endings effect, see Kutas and Federmeier 2011). As the analyses reported above focused on the frontal N400, we conducted additional post hoc analyses on the parietal N400 (see Section S4). Indeed, along with the already reported main effect of N170 on N400 amplitudes, these analyses revealed a condition‐specific effect with more negative N170 amplitudes after delayed negative feedback (indicating unexpected negative feedback being processed in the MTL) predicting more negative N400 amplitudes specifically in the associated target condition (indicating learnt associations). Complementarily, this analysis yielded no significant effects of FRN amplitudes on parietal N400 amplitudes. Based on these post hoc considerations, we would like to put forward the hypothesis that learning‐related brain activity is differentially associated with frontal versus parietal N400 priming effects, which should be replicated in further studies.

Taken together, the observed discrepancy that the FRN predicted specifically association‐driven frontal N400 priming effects and the N170 predicted generally facilitated semantic processing could indicate that procedural and declarative processes affect associative learning differentially (Paller et al. 1995): the fact that higher FRN amplitudes predicted the strength of specifically target‐driven frontal N400 priming effects suggests that these priming effects are linked to procedural learning processes. In contrast, higher N170 amplitudes predicting generally reduced N400 amplitudes (and possible condition‐specific effects for parietal N400 amplitudes) suggest a more in‐depth encoding during learning, possibly favoring recollection of all encountered words irrespective of specifically formed associations. This interpretation finds support in the prediction patterns of the N170 on free recall and recognition performance.

### 3.5 Potential Effects of Feedback Salience

We would like to point out that the deterministic feedback provided in this study led to negative feedback becoming less and less frequent over the course of the learning blocks. This might have increased the salience and thus favored a learning boost for negative feedback at the ends of the learning blocks (i.e., a von‐Restorff‐like effect; Von Restorff 1933). Previous research has shown that feedback salience affects the FRN, with more salient feedback being associated with higher amplitudes (Ferdinand et al. 2012; Oliveira et al. 2007). Although salience effects on the feedback‐locked N170 have not been investigated, familiarity effects on face processing hint at possible influences of salience on the N170 (Caharel and Rossion 2021). Moreover, in the present study, predictive effects of the FRN on recognition performance and of both FRN and N170 on the N400 were specific for negative feedback. To investigate whether these findings could be affected by potential salience‐driven confounds, we performed additional post hoc analyses on the feedback‐locked P300, which is known to reflect salience (Karis et al. 1984, see Section S6). When examining the effect of feedback valence on the feedback‐locked P300 (modeled as dependent variable in this control analysis), we found an enhanced P300 amplitude for negative feedback trials, suggesting a higher salience of these trials. However, when the feedback‐locked P300 was included as predictor of learning outcome, the analyses revealed that higher P300 amplitudes predicted a worse performance in free recall and primed recognition (the latter selectively for immediate feedback), thus speaking against a salience‐driven learning boost. In line with this, lower rather than higher FRN amplitudes predicted better recognition performance and lower N400 amplitudes (while the opposite would be expected if effects were salience‐driven). Overall, this pattern of results suggests that the effects reported in this study were not mainly driven by salience, although a contribution of salience cannot be ruled out.

## 4 Conclusion

In a new study design, we found that feedback‐based learning of novel‐object‐novel‐word associations leads to a strong priming effect, as reflected in the N400. We further found evidence that learning processes represented by the FRN led to more procedural, automatically retrieved memory representations, as indicated by recognition performance, while learning processes represented by the N170 also affected declarative memory representations, as indicated by free recall. With respect to association strength reflected in N400 priming effects, FRN amplitudes predicted the frontal N400 priming effect, while N170 amplitudes predicted generally facilitated semantic processing in the N400 (and condition‐specific effects for parietal N400 amplitudes). No consistent effects of feedback timing were observed, possibly because the learning task with deterministic feedback and the instructions to memorize the novel words facilitated declarative encoding strategies. Feedback valence‐dependent effects of the FRN and N170 can be interpreted in terms of prediction error coding. To confirm these interpretations, a follow‐up study with probabilistic feedback could directly relate trial‐wise prediction error processing to memory performance and association strength.

## Author Contributions

**Christine Albrecht:** conceptualization, data curation, formal analysis, investigation, methodology, software, visualization, writing – original draft, writing – review and editing. **Laura Bechtold:** conceptualization, methodology, validation, writing – original draft, writing – review and editing. **Marta Ghio:** conceptualization, methodology, project administration, validation, writing – review and editing. **Christian Bellebaum:** conceptualization, methodology, project administration, resources, supervision, validation, writing – review and editing.

## Funding

Open Access funding enabled and organized by Projekt DEAL. Author C. Albrecht's position was funded by the Deutsche Forschungsgemeinschaft (DFG; German Research Foundation, project number 467460456) during the conduct of the described study.

## Conflicts of Interest

The authors declare no conflicts of interest.

## Supporting information


**Appendix S1:** psyp70306‐sup‐0001‐AppendixS1.docx.

## Data Availability

The data that support the findings of this study and all analysis scripts are openly accessible through the Open Science Framework at https://doi.org/10.17605/OSF.IO/S36Y2.

## Endnotes

^1^
The amplitude difference between positive and negative feedback has more recently been traced back to a *reward positivity*, which is more pronounced for positive vs. negative feedback (RewP; Proudfit 2015). We from now on use the term FRN to refer to any feedback‐locked negativity in the respective time frame, which should indirectly reflect a reward positivity in relative amplitude differences.
