# Supplementary material for: From Feedback‐Learning to Semantic Memory: Can Feedback‐Related Brain Activity Predict Object‐Word Associations?
Source: Psychophysiology. 2026 May 6;63(5):e70306. doi: 10.1111/psyp.70306 (PMC13148314; doi:10.1111/psyp.70306)
Supplement: Supplementary file 1 — Appendix S1: psyp70306‐sup‐0001‐AppendixS1.docx. [file PSYP-63-e70306-s001.docx]

**SUPPLEMENTARY MATERIAL**

# From Feedback-Learning to Semantic Memory: Can Feedback-Related Brain Activity Predict Object-Word Associations?

**Section S1: Behavioral Results**

***Learning Blocks***

**Accuracy.** In the learning blocks, we found statistically significant main effects of Feedback Timing, *z* = -2.69, *p* = .007, β = -0.31, and Block, *z* = 35.13, *p* < .001, β = 1.77, on accuracy, which were qualified by a significant Feedback Timing by Block interaction, *z* = -6.09, *p* < .001. For both immediate, *z* = 27.81, *p* < .001, β = 2.07, and delayed feedback, *z* = 21.64, *p* < .001, β = 1.46, accuracy increased across blocks, but this effect was stronger for immediate than delayed feedback. The effect is visualized in Figure S1.1 and full inferential statistics are reported in Table S1.1.

**Figure S1.1**

*Mean and single-subject accuracy values across learning blocks*

*
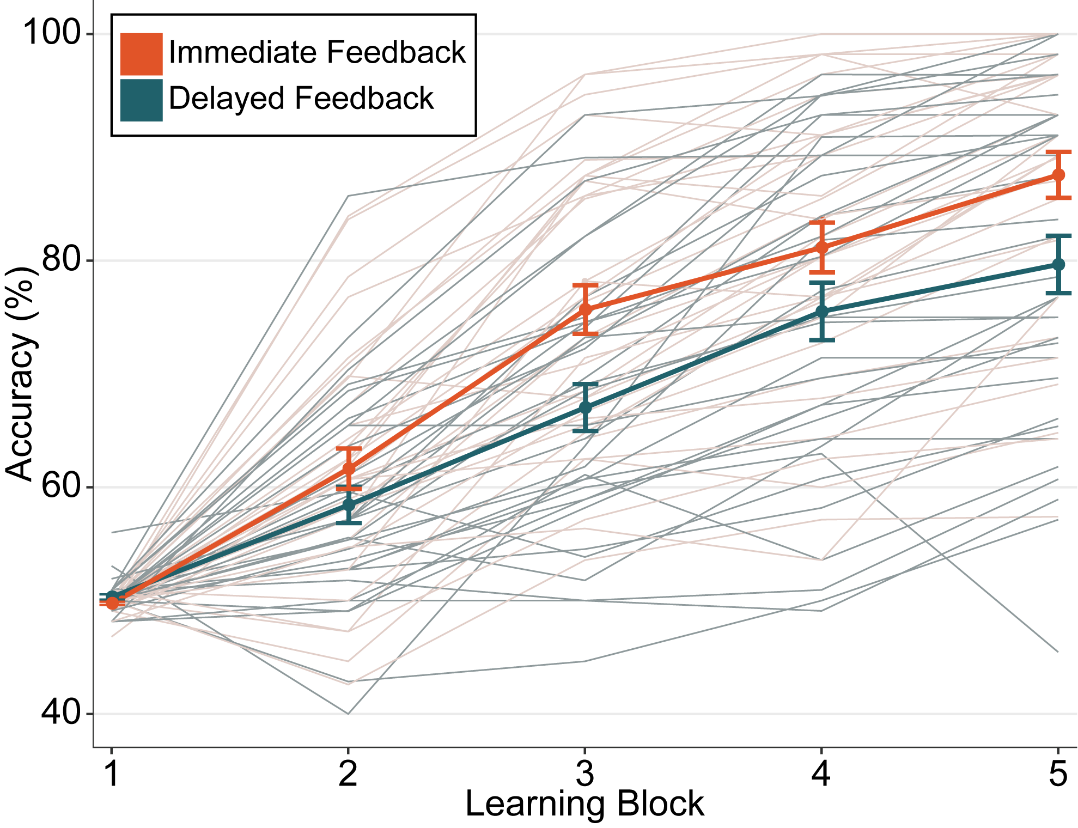
*

*Note.* In the learning blocks, responses were considered correct if participants chose the associated target word. Error bars represent standard errors.

**Table S1.1**

*β-Values, confidence intervals and t-test results for the GLME analysis on accuracy in the learning blocks*

| **Effect** | **β** | ***SE*** | ***z*** | ***p*** | **CI 2.5%** | **CI 97.5%** |
| --- | --- | --- | --- | --- | --- | --- |
| Intercept | 0.90 | 0.06 | 15.75 | **< .001** | 0.79 | 1.02 |
| Feedback Timing | -0.31 | 0.11 | -2.69 | **.007** | -0.53 | -0.05 |
| Block | 1.77 | 0.05 | 35.13 | **< .001** | 1.66 | 1.87 |
| Feedback Timing x Block | -0.61 | 0.10 | -6.09 | **< .001** | -0.83 | -0.42 |

*Note.* The sign of the β-estimates indicates the direction of main effects for the fixed-effects predictors Feedback Timing (immediate [-0.5] vs. delayed [0.5]) and Block (1-5, scaled and mean-centred). GLME = generalized linear mixed effects, β = beta estimate, *SE* = standard error, *CI* = confidence interval of beta estimate.

**Reaction Times.** In the learning blocks, we found statistically significant main effects of Response Correctness, *F*(1,63.92) = 101.99, *p* < .001, β = -0.09, and Block, *F*(1,64.81) = 20.39, *p* < .001, β = -0.12, on reaction times, which were qualified by a significant Response Correctness by Block interaction, *F*(1,13648.77) = 295.00, *p* < .001. Only for correct responses, participants’ reaction time decreased significantly across blocks, *F*(1,65.73) = 75.73, *p* < .001, β = -0.23 (*p* = .778 for wrong responses). No other main or interaction effects were significant (all *p* ≥ .061). A visual display of descriptive results can be found in Figure S1.2, full inferential statistics are reported in Table S1.2.

**Figure S1.2**

*Means and distribution of reaction times across learning blocks*

*
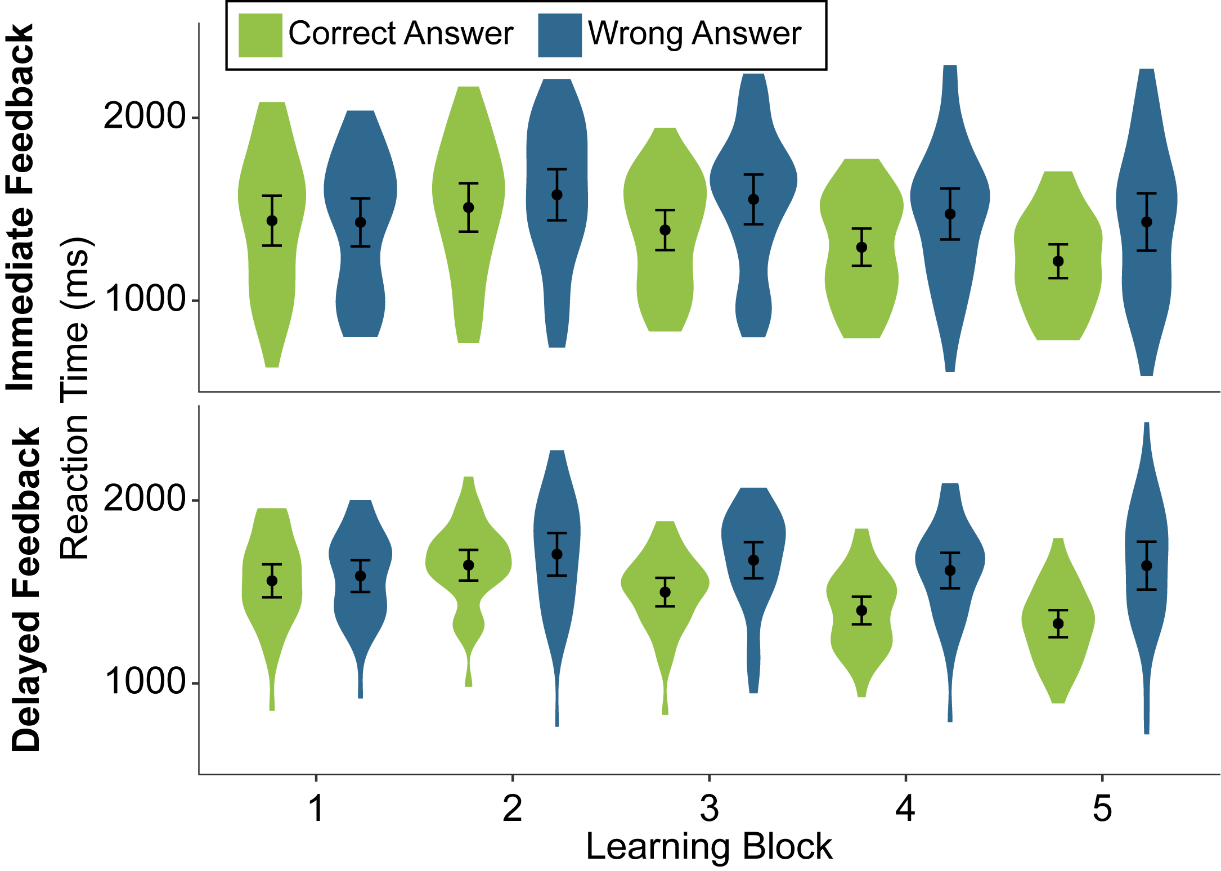
*

*Note.* Dots represent means, error bars represent standard errors. Violin plots represent the distribution across participants.

**Table S1.2**

β*-Values, confidence intervals and t-test results for the LME analysis on log-transformed reaction times in the learning blocks*

| **Effect** | **β** | ***SE*** | **df** | ***t*** | ***p*** | **CI 2.5%** | **CI 97.5%** |
| --- | --- | --- | --- | --- | --- | --- | --- |
| Intercept | 7.25 | 0.03 | 64.17 | 285.73 | < .001 | 7.20 | 7.29 |
| Feedback Timing | 0.10 | 0.05 | 64.17 | 1.90 | .061 | -0.01 | 0.20 |
| Response Correctness | -0.09 | 0.01 | 63.92 | -10.10 | < .001 | -0.11 | -0.07 |
| Block | -0.12 | 0.03 | 64.81 | -4.52 | < .001 | -0.17 | -0.06 |
| Feedback Timing x Response Correctness | -0.02 | 0.02 | 63.92 | -0.92 | .359 | -0.05 | 0.02 |
| Feedback Timing x Block | -0.03 | 0.05 | 64.81 | -0.60 | .550 | -0.13 | 0.06 |
| Response Correctness x Block | -0.22 | 0.01 | 13648.77 | -17.18 | < .001 | -0.24 | -0.19 |
| Feedback Timing x Response Correctness x Block | 0.03 | 0.03 | 13648.77 | 1.12 | .262 | -0.02 | 0.08 |

*Note.* The sign of the β-estimates indicates the direction of main effects for the fixed-effects predictors Feedback Timing (immediate [-0.5] vs. delayed [0.5]), and Response Correctness (wrong [-0.5] vs. correct [0.5]) and Block (1-5, scaled and mean-centred). LME = linear mixed effects, β = beta estimate, *SE* = standard error, *CI* = confidence interval of beta estimate.

***Free Recall Test and Primed Recognition Task***

**Accuracy.** Table S1.3 reports descriptive accuracy in the free recall test and primed recognition task.

**Table S1.3**

*Accuracy in the free recall test and primed recognition task*

| Test Part | Feedback Timing | *M* | *SD* | *Min* | *Max* |
| --- | --- | --- | --- | --- | --- |
| Free Recall | Immediate | 48.21 | 27.51 | 0.00 | 98.21 |
|  | Delayed | 41.46 | 26.00 | 0.00 | 98.21 |
| Primed Recognition | Immediate | 55.14 | 26.66 | 0.00 | 98.21 |
|  | Delayed | 50.02 | 25.85 | 3.41 | 94.60 |

*Note. M* = mean, *SD* = standard deviation, *Min* = minimum value, *Max* = maximum value. In the free recall test, responses were considered correct if participants entered the associated target word (allowing for deviations of one character). In the primed recognition task, responses were considered correct if participants correctly accepted the associated target novel word and correctly rejected the three unrelated novel words (i.e., the associated distractor, the not associated target and the not associated distractor words).

**Reaction Times in the Primed Recognition Task.** In the primed recognition task, reaction times were significantly influenced by Response Correctness, *F*(1,53.95) = 87.29, *p* < .001, β = -0.18, which was further explained by a significant interaction between Response Correctness and Priming Condition, *F*(3,13974.83) = 17.79, *p* < .001. For all levels of Priming Condition, i.e., associated target (*p* < .001, β = -0.10), associated distractor (*p* < .001, β = -0.20), not associated target (*p* < .001, β = -0.19) and not associated distractor (*p* < .001, β = -0.22), reaction times were significantly faster for correct responses, with the smallest Response Correctness effect occurring for the associated target condition. No other effects were significant (all *p* ≥ .130). For descriptive results, refer to Figure S1.3. Full inferential statistics are reported in Table S1.4.

**Figure S1.3**

*Means and distribution of reaction times in the primed recognition task*


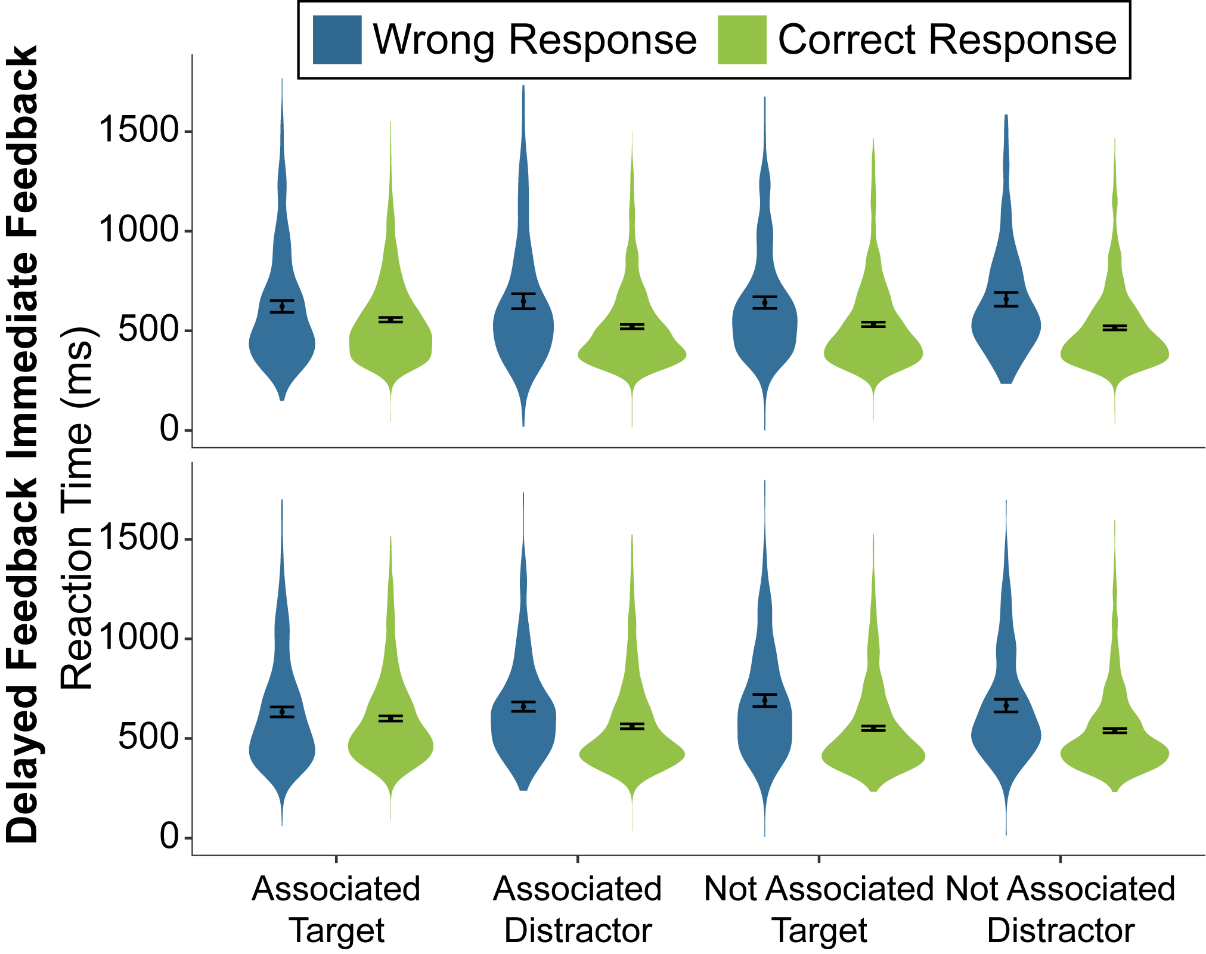


*Note.* Dots represent means, error bars represent standard errors. Violin plots represent the distribution across participants.

**Table S1.4**

β*-Values, confidence intervals and t-test results for the LME analysis on log-transformed reaction times in the primed recognition task*

| **Effect** | **β** | ***SE*** | **df** | ***t*** | ***p*** | **CI 2.5%** | **CI 97.5%** |
| --- | --- | --- | --- | --- | --- | --- | --- |
| Intercept | 6.35 | 0.02 | 62.60 | 259.85 | < .001 | 6.30 | 6.39 |
| Feedback Timing | 0.04 | 0.05 | 62.60 | 0.85 | .400 | -0.04 | 0.14 |
| Associated Distractor | -0.02 | 0.01 | 13991.20 | -1.76 | .079 | -0.04 | 0.00 |
| Not Associated Distractor | -0.02 | 0.01 | 13933.28 | -2.21 | .027 | -0.04 | 0.00 |
| Not Associated Target | -0.01 | 0.01 | 13879.25 | -1.25 | .213 | -0.03 | 0.01 |
| Response Correctness | -0.18 | 0.02 | 53.95 | -9.34 | < .001 | -0.22 | -0.14 |
| Feedback Timing x Associated Distractor | 0.01 | 0.02 | 13991.20 | 0.47 | .641 | -0.03 | 0.04 |
| Feedback Timing x Not Associated Distractor | -0.03 | 0.02 | 13933.28 | -1.25 | .211 | -0.06 | 0.02 |
| Feedback Timing x Not Associated Target | -0.01 | 0.02 | 13879.25 | -0.34 | .737 | -0.04 | 0.03 |
| Feedback Timing x Response Correctness | 0.04 | 0.04 | 53.95 | 1.10 | .277 | -0.03 | 0.12 |
| Associated Distractor x Response Correctness | -0.10 | 0.02 | 13984.46 | -5.49 | < .001 | -0.14 | -0.07 |
| Not Associated Distractor x Response Correctness | -0.13 | 0.02 | 13979.21 | -6.44 | < .001 | -0.17 | -0.09 |
| Not Associated Target x Response Correctness | -0.10 | 0.02 | 13961.46 | -5.31 | < .001 | -0.14 | -0.06 |
| Feedback Timing x Associated Distractor x Response Correctness | 0.01 | 0.04 | 13984.46 | 0.36 | .717 | -0.06 | 0.08 |
| Feedback Timing x Not Associated Distractor x Response Correctness | 0.03 | 0.04 | 13979.21 | 0.65 | .518 | -0.05 | 0.10 |
| Feedback Timing x Not Associated Target x Response Correctness | -0.05 | 0.04 | 13961.46 | -1.20 | .231 | -0.12 | 0.03 |

*Note.* The sign of the β-estimates indicates the direction of main effects for the fixed-effects predictors Feedback Timing (immediate [-0.5] vs. delayed [0.5]), Priming Condition (baseline = associated target, compared to associated distractor, not associated distractor, and not associated target) and Response Correctness (wrong [-0.5] vs. correct [0.5]). LME = linear mixed effects, β = beta estimate, *SE* = standard error, *CI* = confidence interval of beta estimate.

**Section S2: Number of Data Points**

The preprocessing and data analysis procedures included three steps in which potential outlier trials were removed. In a first step, trials in which participants failed to respond were excluded. In a second step, an automatic artifact rejection (see EEG data preprocessing section in the manuscript) was conducted. During statistical analysis, residual outliers were then removed based on the ERPs included in the respective analyses (see Statistical Data Analysis section in the manuscript). For a full picture of removed datapoints, we report the average initial number of datapoints for participants before any removals (Table S2.1), the number of correct responses, incorrect responses, and misses in each experimental task in which ERPs were derived (Table S2.2), and the number of remaining data points after automatic artifact rejection (Table S2.2). In Section S3, we also report the included number of datapoints for each analysis after residual outlier removal.

**Table S2.1**

*Number of datapoints per participant for all trial types before any removals*

| Condition | *M* | *SD* | *Min* | *Max* |
| --- | --- | --- | --- | --- |
| Positive Feedback | 190.29 | 26.41 | 137 | 240 |
| Negative Feedback | 86.39 | 25.40 | 39 | 143 |
| Associated Target | 56 | 0 | 56 | 56 |
| Associated Distractor | 56 | 0 | 56 | 56 |
| Non-Associated Target | 56 | 0 | 56 | 56 |
| Non-Associated Distractor | 56 | 0 | 56 | 56 |

*Note. M* = Mean, *SD* = Standard Deviation, *Min* = Minimum, *Max* = Maximum.

**Table S2.2**

*Number of datapoints per participant by response type for all parts of the experiment in which*

*ERPs were derived*

|  | Correct Response | | Wrong Response | | Misses | |
| --- | --- | --- | --- | --- | --- | --- |
|  | *M*  (*SD*) | *Min-Max* | *M*  (*SD*) | *Min-Max* | *M*  (*SD*) | *Min-Max* |
| Learning Blocks | 190.29  (26.41) | 137-240 | 86.39  (25.40) | 39-143 | 2.36  (2.24) | 0-10 |
| Associated Target | 41.98  (8.85) | 17-56 | 13.95  (8.83) | 0-39 | 0.06  (0.39) | 0-3 |
| Associated Distractor | 44.30  (9.34) | 20-56 | 11.61  (9.26) | 0-36 | 0.09  (0.34) | 0-2 |
| Non-Associated Target | 45.00  (10.76) | 6-56 | 10.88  (10.75) | 0-50 | 0.12  (0.41) | 0-2 |
| Non-Associated Distractor | 47.46  (9.67) | 4-56 | 8.44  (9.63) | 0-52 | 0.09  (0.34) | 0-2 |

*Note. M* = Mean, *SD* = Standard Deviation, *Min* = Minimum, *Max* = Maximum.

**Table S2.3**

*Number of datapoints per participant for all trial types after artifact rejection*

| Condition | *M* | *SD* | *Min* | *Max* |
| --- | --- | --- | --- | --- |
| Positive Feedback | 184.08 | 26.80 | 126 | 240 |
| Negative Feedback | 83.71 | 24.78 | 36 | 138 |
| Associated Target | 54.53 | 2.37 | 45 | 56 |
| Associated Distractor | 54.53 | 2.32 | 45 | 56 |
| Non-Associated Target | 54.38 | 2.46 | 45 | 56 |
| Non-Associated Distractor | 54.44 | 2.25 | 46 | 56 |

*Note. M* = Mean, *SD* = Standard Deviation, *Min* = Minimum, *Max* = Maximum.

**Section S3: Results - Full Inferential Statistics**

For better readability, in the main text we only reported significant effects (based on an α = .05). Full inferential statistics, including t-values/z-values, β-values, confidence intervals of β-values, as well as the mean number of data points per condition included in the respective analysis for all models can be found in the following Tables S3.1 – S3.14**.**

**Control Analyses**

***FRN***

| **Table S3.1**  *Number of datapoints per participant and condition for the FRN control analysis, Recall-FRN Analysis and Recognition-FRN analysis*   \| Feedback Timing \| Feedback Valence \| *M* \| *SD* \| *Min* \| *Max* \| \| --- \| --- \| --- \| --- \| --- \| --- \| \| Immediate Feedback \| Positive Feedback \| 184.00 \| 26.12 \| 140 \| 240 \| \| Negative Feedback \| 73.94 \| 23.13 \| 39 \| 128 \| \| Delayed Feedback \| Positive Feedback \| 165.48 \| 25.87 \| 120 \| 215 \| \| Negative Feedback \| 85.42 \| 24.37 \| 33 \| 136 \|   *Note. M* = Mean, *SD* = Standard Deviation, *Min* = Minimum, *Max* = Maximum  **Table S3.2**  β*-Values, confidence intervals and t-test results for the LME analysis on FRN amplitude* | | | | | | | |  |
| --- | --- | --- | --- | --- | --- | --- | --- | --- | --- | --- | --- | --- | --- | --- | --- | --- | --- | --- | --- | --- | --- | --- | --- | --- | --- | --- | --- | --- | --- | --- | --- | --- | --- | --- | --- | --- |
| **Effects** | **β** | ***SE*** | ***df*** | ***t*** | ***p*** | ***2.5% CI*** | ***97.5% CI*** | |
| Intercept | -3.71 | 0.27 | 63.99 | -13.90 | **< .001** | -4.21 | -3.18 | |
| Feedback Timing | 0.31 | 0.53 | 63.99 | 0.58 | .564 | -0.73 | 1.40 | |
| Feedback Valence | 1.30 | 0.20 | 63.51 | 6.49 | **< .001** | 0.92 | 1.65 | |
| Feedback Timing x Feedback Valence | -1.60 | 0.40 | 63.51 | -4.00 | **< .001** | -2.40 | -0.83 | |
| *Note.* The sign of the β-estimates indicates the direction of main effects for the fixed-effects predictors Feedback Timing (immediate [-0.5] vs. delayed [0.5]), and Feedback Valence (negative [-0.5] vs. positive [0.5]). LME = linear mixed effects, FRN = feedback-related negativity, β = beta estimate, *SE* = standard error, *CI* = confidence interval of beta estimate. | | | | | | | | |

***N170***

| **Table S3.3**  *Number of datapoints per participant and condition for the N170 control analysis, Recall-N170 analysis and Recognition-N170 analysis*   \| Feedback Timing \| Feedback Valence \| *M* \| *SD* \| *Min* \| *Max* \| \| --- \| --- \| --- \| --- \| --- \| --- \| \| Immediate Feedback \| Positive Feedback \| 184.12 \| 26.48 \| 139 \| 238 \| \| Negative Feedback \| 75.58 \| 22.99 \| 39 \| 126 \| \| Delayed Feedback \| Positive Feedback \| 166.21 \| 28.64 \| 116 \| 216 \| \| Negative Feedback \| 83.91 \| 23.73 \| 35 \| 136 \|   *Note. M* = Mean, *SD* = Standard Deviation, *Min* = Minimum, *Max* = Maximum.  **Table S3.4**  β*-Values, confidence intervals and t-test results for the LME analysis on N170 amplitude* | | | | | | | |  |
| --- | --- | --- | --- | --- | --- | --- | --- | --- | --- | --- | --- | --- | --- | --- | --- | --- | --- | --- | --- | --- | --- | --- | --- | --- | --- | --- | --- | --- | --- | --- | --- | --- | --- | --- | --- | --- |
| **Effects** | **β** | ***SE*** | ***df*** | ***t*** | ***p*** | ***2.5% CI*** | ***97.5% CI*** | |
| Intercept | -6.62 | 0.37 | 63.89 | -17.90 | **< .001** | -7.26 | -5.94 | |
| Feedback Timing | 0.02 | 0.74 | 63.89 | 0.02 | .983 | -1.55 | 1.45 | |
| Feedback Valence | 1.55 | 0.16 | 65.09 | 9.94 | **< .001** | 1.24 | 1.83 | |
| Feedback Timing x Feedback Valence | -0.83 | 0.31 | 65.09 | -2.67 | **.010** | -1.42 | -0.21 | |
| *Note.* The sign of the β-estimates indicates the direction of main effects for the fixed-effects predictors Feedback Timing (immediate [-0.5] vs. delayed [0.5]), and Feedback Valence (negative [-0.5] vs. positive [0.5]). LME = linear mixed effects, β = beta estimate, *SE* = standard error, *CI* = confidence interval of beta estimate. | | | | | | | | |

**Main Analyses**

**Figure S3.1**

*Feedback-locked grand average ERP waveforms for the FRN (pooled over Fz, FCz, and Cz) and N170 (pooled over P7 and P8) by Feedback Timing, Feedback Valence and Recall/Recognition accuracy*

*
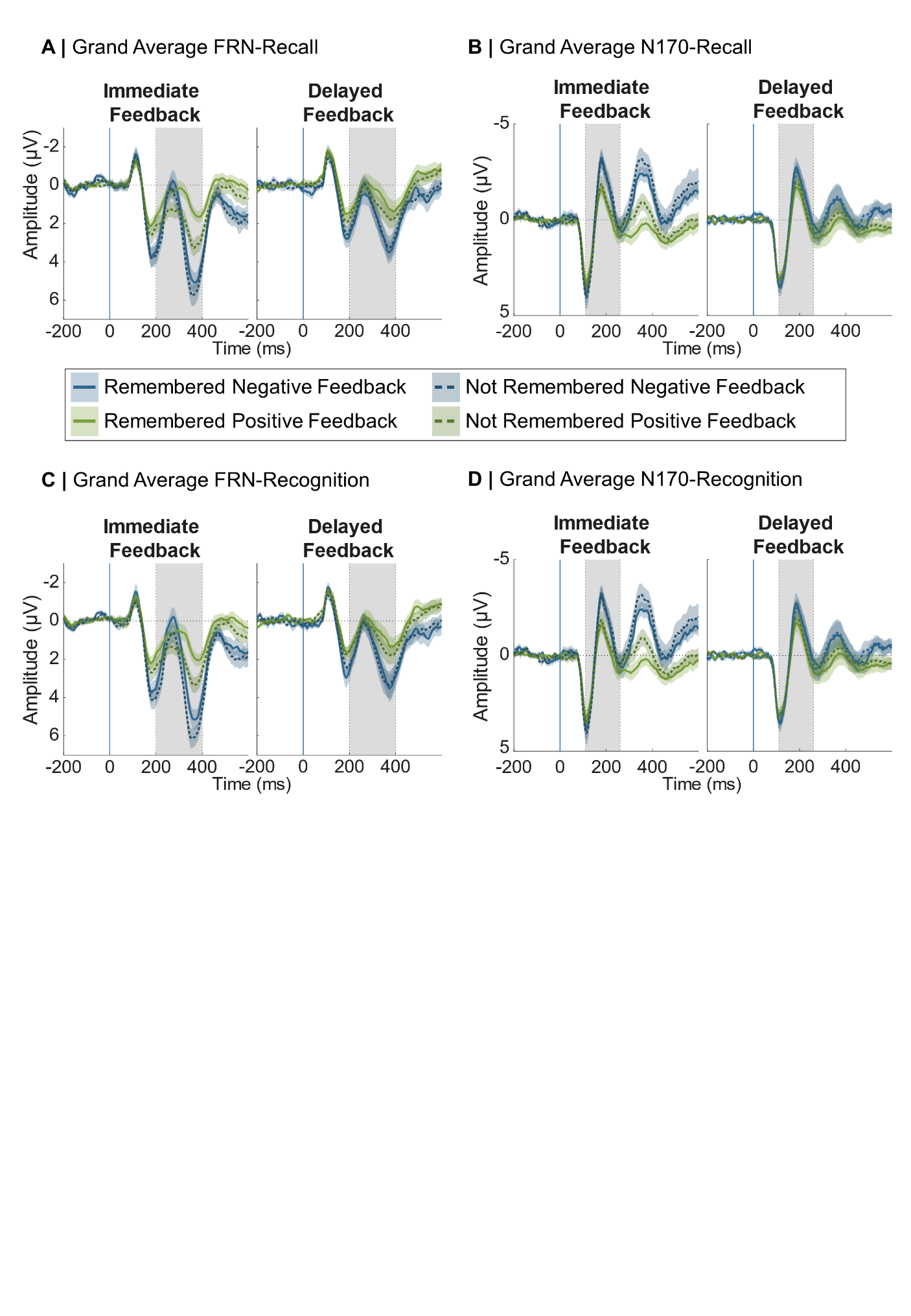
*

*Note.* Error margins around the grand averages (*n* = 33 in both Feedback Delay conditions) represent ± 1 standard error. Gray areas represent the time window in which the maximum negative peaks per condition and participant were searched. Please note that grand averages reflect the mean across participants at any given timepoint, and might therefore not directly correspond to results obtained with the generalized linear effects models. These models were based on peak-to-peak single trial amplitudes computed by taking into account subject- and condition-wise latencies, included random slopes and intercepts by participant, excluded residual outliers and accounted for group sizes.

***Prediction of Recall Performance***

**FRN-Recall.**

| **Table S3.5**  β*-Values, confidence intervals and t-test results for the GLME analysis on free recall accuracy including FRN as predictor* | | | | | | |  |
| --- | --- | --- | --- | --- | --- | --- | --- |
| **Effects** | **β** | ***SE*** | ***z*** | ***p*** | ***2.5% CI*** | ***97.5% CI*** | |
| Intercept | -0.41 | 0.23 | -1.77 | .076 | -0.84 | 0.03 | |
| Feedback Timing | -0.37 | 0.46 | -0.81 | .417 | -1.14 | 0.52 | |
| FRN Amplitude | 0.29 | 0.16 | 1.83 | .067 | -0.04 | 0.63 | |
| Feedback Timing x FRN Amplitude | -0.56 | 0.30 | -1.85 | .064 | -1.23 | 0.09 | |
| FRN Amplitude x Feedback Valence | -0.45 | 0.36 | -1.25 | .211 | -1.26 | 0.30 | |
| Feedback Timing x FRN Amplitude x Feedback Valence | 1.35 | 0.70 | 1.92 | .055 | -0.13 | 2.84 | |
| *Note.* The sign of the β-estimates indicates the direction of main effects for the fixed-effects predictors Feedback Timing (immediate [-0.5] vs. delayed [0.5]), FRN Amplitude (scaled and mean-centered), and Feedback Valence (nested in FRN Amplitude, negative [-0.5] vs. positive [0.5]). GLME: generalized linear mixed effects, β = beta estimate, *SE* = standard error, *CI* = confidence interval of beta estimate. | | | | | | | |

**N170-Recall.**

| **Table S3.6**  β*-Values, confidence intervals and t-test results for the GLME analysis on free recall accuracy including N170 as predictor* | | | | | | |  |
| --- | --- | --- | --- | --- | --- | --- | --- |
| **Effects** | **β** | ***SE*** | ***z*** | ***p*** | ***2.5% CI*** | ***97.5% CI*** | |
| Intercept | -0.43 | 0.23 | -1.86 | .063 | -0.85 | 0.03 | |
| Feedback Timing | -0.34 | 0.46 | -0.74 | .461 | -1.21 | 0.64 | |
| N170 Amplitude | 1.16 | 0.19 | 6.06 | **< .001** | 0.74 | 1.55 | |
| Feedback Timing x N170 Amplitude | -1.11 | 0.36 | -3.09 | **.002** | -1.85 | -0.29 | |
| N170 Amplitude x Feedback Valence | 0.39 | 0.51 | 0.78 | .437 | -0.65 | 1.43 | |
| Feedback Timing x N170 Amplitude x Feedback Valence | 0.27 | 0.98 | 0.27 | .787 | -1.89 | 2.01 | |
| *Note.* The sign of the β-estimates indicates the direction of main effects for the fixed-effects predictors Feedback Timing (immediate [-0.5] vs. delayed [0.5]), N170 Amplitude (scaled and mean-centered), and Feedback Valence (nested in FRN Amplitude, negative [-0.5] vs. positive [0.5]). GLME: generalized linear mixed effects, β = beta estimate, *SE* = standard error, *CI* = confidence interval of beta estimate. | | | | | | | |

***Prediction of Recognition Performance***

**FRN-Recognition.**

| **Table S3.7**  β*-Values, confidence intervals and t-test results for the GLME analysis on recognition accuracy including FRN as predictor* | | | | | | |  |
| --- | --- | --- | --- | --- | --- | --- | --- |
| **Effects** | **β** | ***SE*** | ***z*** | ***p*** | ***2.5% CI*** | ***97.5% CI*** | |
| Intercept | 0.10 | 0.19 | 0.53 | .599 | -0.28 | 0.45 | |
| Feedback Timing | -0.27 | 0.38 | -0.71 | .477 | -0.94 | 0.48 | |
| FRN Amplitude | 0.26 | 0.13 | 2.05 | **.041** | 0.02 | 0.53 | |
| Feedback Timing x FRN Amplitude | -0.11 | 0.25 | -0.44 | .657 | -0.68 | 0.39 | |
| FRN Amplitude x Feedback Valence | -0.59 | 0.24 | -2.49 | **.013** | -1.08 | -0.15 | |
| Feedback Timing x FRN Amplitude x Feedback Valence | 0.22 | 0.47 | 0.47 | .639 | -0.73 | 1.17 | |
| *Note.* The sign of the β-estimates indicates the direction of main effects for the fixed-effects predictors Feedback Timing (immediate [-0.5] vs. delayed [0.5]), FRN Amplitude (scaled and mean-centered), and Feedback Valence (nested in FRN Amplitude, negative [-0.5] vs. positive [0.5]). GLME: generalized linear mixed effects, β = beta estimate, *SE* = standard error, *CI* = confidence interval of beta estimate. | | | | | | | |

**N170-Recognition.**

| **Table S3.8**  β*-Values, confidence intervals and t-test results for the GLME analysis on recognition accuracy including N170 as predictor* | | | | | | |  |
| --- | --- | --- | --- | --- | --- | --- | --- |
| **Effects** | **β** | ***SE*** | ***z*** | ***p*** | ***2.5% CI*** | ***97.5% CI*** | |
| Intercept | 0.09 | 0.19 | 0.44 | .657 | -0.25 | 0.46 | |
| Feedback Timing | -0.25 | 0.39 | -0.65 | .514 | -1.07 | 0.52 | |
| N170 Amplitude | 1.13 | 0.19 | 5.84 | **< .001** | 0.77 | 1.49 | |
| Feedback Timing x N170 Amplitude | -0.85 | 0.37 | -2.29 | **.022** | -1.68 | -0.14 | |
| N170 Amplitude x Feedback Valence | 0.50 | 0.45 | 1.13 | .259 | -0.44 | 1.34 | |
| Feedback Timing x N170 Amplitude x Feedback Valence | 0.40 | 0.88 | 0.46 | .644 | -1.36 | 2.17 | |
| *Note.* The sign of the β-estimates indicates the direction of main effects for the fixed-effects predictors Feedback Timing (immediate [-0.5] vs. delayed [0.5]), N170 Amplitude (scaled and mean-centered), and Feedback Valence (nested in FRN Amplitude, negative [-0.5] vs. positive [0.5]). GLME: generalized linear mixed effects, β = beta estimate, *SE* = standard error, *CI* = confidence interval of beta estimate. | | | | | | | |

***N400 priming effect***

**Table S3.9**

*Number of datapoints per participant and condition for the N400 priming effect analysis*

| Recognition | Priming Condition | Laterality | Frontality | *M* | *SD* | *Min* | *Max* |
| --- | --- | --- | --- | --- | --- | --- | --- |
| remembered | associated target | left | frontal | 263.43 | 134.44 | 10 | 528 |
|  |  |  | parietal | 270.34 | 137.83 | 18 | 533 |
|  |  | central | frontal | 261.45 | 133.45 | 10 | 538 |
|  |  |  | parietal | 134.72 | 69.06 | 9 | 264 |
|  |  | right | frontal | 261.69 | 133.18 | 18 | 538 |
|  |  |  | parietal | 271.15 | 137.58 | 18 | 533 |
|  | associated distractor | left | frontal | 263.60 | 132.51 | 14 | 533 |
|  |  |  | parietal | 269.85 | 135.45 | 18 | 548 |
|  |  | central | frontal | 260.48 | 131.61 | 15 | 548 |
|  |  |  | parietal | 134.40 | 66.67 | 9 | 269 |
|  |  | right | frontal | 261.80 | 133.17 | 18 | 548 |
|  |  |  | parietal | 269.49 | 135.80 | 18 | 543 |
|  | not associated target | left | frontal | 266.57 | 135.83 | 13 | 543 |
|  |  |  | parietal | 271.18 | 138.67 | 13 | 543 |
|  |  | central | frontal | 265.62 | 136.83 | 15 | 548 |
|  |  |  | parietal | 134.72 | 68.26 | 9 | 259 |
|  |  | right | frontal | 264.42 | 135.42 | 13 | 543 |
|  |  |  | parietal | 270.88 | 136.97 | 9 | 533 |
|  | not associated distractor | left | frontal | 267.49 | 133.02 | 13 | 523 |
|  |  |  | parietal | 273.88 | 136.86 | 18 | 548 |
|  |  | central | frontal | 266.17 | 134.05 | 18 | 548 |
|  |  |  | parietal | 134.77 | 67.49 | 9 | 274 |
|  |  | right | frontal | 265.82 | 133.52 | 13 | 543 |
|  |  |  | parietal | 271.85 | 135.99 | 18 | 548 |

| Recognition | Priming Condition | Laterality | Frontality | *M* | *SD* | *Min* | *Max* |
| --- | --- | --- | --- | --- | --- | --- | --- |
| not remembered | associated target | left | frontal | 232.74 | 128.98 | 10 | 520 |
|  |  |  | parietal | 236.64 | 132.76 | 10 | 540 |
|  |  | central | frontal | 230.33 | 129.71 | 10 | 473 |
|  |  |  | parietal | 117.85 | 66.18 | 5 | 265 |
|  |  | right | frontal | 230.86 | 130.78 | 10 | 521 |
|  |  |  | parietal | 238.82 | 133.85 | 10 | 545 |
|  | associated distractor | left | frontal | 232.56 | 128.45 | 10 | 507 |
|  |  |  | parietal | 237.29 | 132.15 | 10 | 550 |
|  |  | central | frontal | 229.98 | 127.21 | 10 | 501 |
|  |  |  | parietal | 118.14 | 66.99 | 5 | 270 |
|  |  | right | frontal | 232.94 | 130.60 | 10 | 526 |
|  |  |  | parietal | 239.12 | 133.30 | 10 | 545 |
|  | not associated target | left | frontal | 232.83 | 130.38 | 10 | 515 |
|  |  |  | parietal | 236.14 | 133.17 | 10 | 550 |
|  |  | central | frontal | 228.38 | 128.14 | 10 | 500 |
|  |  |  | parietal | 119.45 | 67.07 | 5 | 275 |
|  |  | right | frontal | 231.59 | 131.61 | 10 | 506 |
|  |  |  | parietal | 237.58 | 133.59 | 10 | 550 |
|  | not associated distractor | left | frontal | 231.88 | 128.62 | 10 | 525 |
|  |  |  | parietal | 236.20 | 131.90 | 10 | 550 |
|  |  | central | frontal | 229.06 | 126.75 | 10 | 496 |
|  |  |  | parietal | 117.50 | 65.49 | 5 | 264 |
|  |  | right | frontal | 228.85 | 129.12 | 10 | 486 |
|  |  |  | parietal | 236.71 | 132.12 | 10 | 546 |

*Note. M* = Mean, *SD* = Standard Deviation, *Min* = Minimum, *Max* = Maximum.

| **Table S3.10**  β*-Values, confidence intervals and t-test results for the LME analysis on N400 amplitude (N400 priming effect)* | | | | | | | |  |
| --- | --- | --- | --- | --- | --- | --- | --- | --- |
| **Effects** | **β** | ***SE*** | ***df*** | ***t*** | ***p*** | ***2.5% CI*** | ***97.5% CI*** | |
| Intercept | 0.69 | 0.40 | 6.25 | 1.73 | .133 | -0.17 | 1.45 | |
| Recognition Accuracy | 0.08 | 0.01 | 720062.39 | 7.53 | **< .001** | 0.06 | 0.10 | |
| Associated Distractor | -0.40 | 0.01 | 721144.54 | -30.13 | **< .001** | -0.42 | -0.37 | |
| Not Associated Distractor | -0.52 | 0.01 | 721143.92 | -38.99 | **< .001** | -0.54 | -0.49 | |
| Not Associated Target | -0.44 | 0.01 | 721144.15 | -33.39 | **< .001** | -0.47 | -0.42 | |
| Left | -0.15 | 0.96 | 5.11 | -0.15 | .885 | -2.13 | 1.60 | |
| Right | -0.21 | 0.96 | 5.13 | -0.22 | .834 | -2.18 | 1.57 | |
| Frontality | 1.04 | 0.75 | 4.99 | 1.38 | .227 | -0.36 | 2.48 | |
| Recognition Accuracy x Associated Distractor | -0.80 | 0.03 | 721144.05 | -29.94 | **< .001** | -0.85 | -0.75 | |
| Recognition Accuracy x Not Associated Distractor | -0.83 | 0.03 | 721143.92 | -31.27 | **< .001** | -0.88 | -0.78 | |
| Recognition Accuracy x Not Associated Target | -0.78 | 0.03 | 721144.10 | -29.55 | **< .001** | -0.84 | -0.74 | |
| Recognition Accuracy x Left | 0.08 | 0.03 | 477406.73 | 2.97 | **.003** | 0.03 | 0.14 | |
| Recognition Accuracy x Right | 0.16 | 0.03 | 486724.35 | 5.86 | **< .001** | 0.11 | 0.22 | |
| Associated Distractor x Left | 0.24 | 0.03 | 721148.59 | 7.27 | **< .001** | 0.18 | 0.31 | |
| Not Associated Distractor x Right | 0.31 | 0.03 | 721146.26 | 9.21 | **< .001** | 0.23 | 0.38 | |
| Not Associated Target x Left | 0.24 | 0.03 | 721146.92 | 7.06 | **< .001** | 0.17 | 0.30 | |
| Associated Distractor x Right | 0.07 | 0.03 | 721148.64 | 2.13 | **.033** | 0.00 | 0.13 | |
| Not Associated Distractor x Left | 0.09 | 0.03 | 721146.07 | 2.69 | **.007** | 0.02 | 0.16 | |
| Not Associated Target x Right | 0.03 | 0.03 | 721147.57 | 0.95 | .342 | -0.03 | 0.09 | |
| Recognition Accuracy x Frontality | 0.43 | 0.02 | 721145.68 | 22.91 | **< .001** | 0.39 | 0.47 | |
| Associated Distractor x Frontality | -0.24 | 0.03 | 721143.97 | -8.85 | **< .001** | -0.28 | -0.18 | |
| Not Associated Distractor x Frontality | -0.06 | 0.03 | 721143.74 | -2.36 | **.018** | -0.12 | -0.01 | |
| Not Associated Target x Frontality | -0.13 | 0.03 | 721143.90 | -4.78 | **< .001** | -0.18 | -0.08 | |
| Left x Frontality | 0.00 | 1.91 | 5.00 | 0.00 | .999 | -3.61 | 3.47 | |
| Right x Frontality | 0.91 | 1.91 | 5.00 | 0.47 | .655 | -2.91 | 4.87 | |

| **Effects** | **β** | ***SE*** | ***df*** | ***t*** | ***p*** | ***2.5% CI*** | ***97.5% CI*** |
| --- | --- | --- | --- | --- | --- | --- | --- |
| Recognition Accuracy x Associated Distractor x Left | 0.37 | 0.07 | 721148.94 | 5.51 | **< .001** | 0.24 | 0.51 |
| Recognition Accuracy x Not Associated Distractor x Left | 0.58 | 0.07 | 721148.53 | 8.60 | **< .001** | 0.44 | 0.72 |
| Recognition Accuracy x Not Associated Target x Left | 0.54 | 0.07 | 721147.46 | 8.01 | **< .001** | 0.41 | 0.68 |
| Recognition Accuracy x Associated Distractor x Right | 0.20 | 0.07 | 721148.90 | 2.99 | **.003** | 0.07 | 0.34 |
| Recognition Accuracy x Not Associated Distractor x Right | 0.41 | 0.07 | 721147.88 | 6.15 | **< .001** | 0.28 | 0.55 |
| Recognition Accuracy x Not Associated Target x Right | 0.11 | 0.07 | 721147.65 | 1.60 | .109 | -0.03 | 0.26 |
| Recognition Accuracy x Associated Distractor x Frontality | -0.36 | 0.05 | 721143.45 | -6.79 | **< .001** | -0.47 | -0.25 |
| Recognition Accuracy x Not Associated Distractor x Frontality | -0.42 | 0.05 | 721143.66 | -7.94 | **< .001** | -0.52 | -0.31 |
| Recognition Accuracy x Not Associated Target x Frontality | -0.62 | 0.05 | 721143.70 | -11.61 | **< .001** | -0.71 | -0.51 |
| Recognition Accuracy x Left x Frontality | -0.12 | 0.05 | 721147.36 | -2.48 | **.013** | -0.22 | -0.03 |
| Recognition Accuracy x Right x Frontality | -0.26 | 0.05 | 721146.27 | -5.45 | **< .001** | -0.36 | -0.17 |
| Associated Distractor x Left x Frontality | 0.45 | 0.07 | 721143.88 | 6.69 | **< .001** | 0.33 | 0.58 |
| Not Associated Distractor x Right x Frontality | 0.42 | 0.07 | 721144.18 | 6.30 | **< .001** | 0.29 | 0.57 |
| Not Associated Target x Left x Frontality | 0.58 | 0.07 | 721144.18 | 8.65 | **< .001** | 0.46 | 0.70 |
| Associated Distractor x Right x Frontality | 0.22 | 0.07 | 721144.09 | 3.24 | **.001** | 0.10 | 0.35 |
| Not Associated Distractor x Left x Frontality | 0.04 | 0.07 | 721143.75 | 0.61 | .542 | -0.08 | 0.18 |
| Not Associated Target x Right x Frontality | 0.23 | 0.07 | 721144.28 | 3.48 | **.001** | 0.10 | 0.37 |
| Recognition Accuracy x Associated Distractor x Left x Frontality | 0.61 | 0.13 | 721143.61 | 4.52 | **< .001** | 0.34 | 0.84 |
| Recognition Accuracy x Not Associated Distractor x Left x Frontality | 0.68 | 0.13 | 721143.77 | 5.04 | **< .001** | 0.38 | 0.95 |
| Recognition Accuracy x Not Associated Target x Left x Frontality | 0.80 | 0.13 | 721144.39 | 5.94 | **< .001** | 0.53 | 1.07 |
| Recognition Accuracy x Associated Distractor x Right x Frontality | 0.05 | 0.13 | 721143.76 | 0.39 | .693 | -0.21 | 0.33 |
| Recognition Accuracy x Not Associated Distractor x Right x Frontality | 0.55 | 0.13 | 721143.69 | 4.05 | **< .001** | 0.29 | 0.81 |
| Recognition Accuracy x Not Associated Target x Right x Frontality | 0.50 | 0.13 | 721144.68 | 3.68 | **< .001** | 0.22 | 0.77 |
| *Note.* The sign of the β-estimates indicates the direction of main effects for the fixed-effects predictors Priming Condition (simple-coding matrix with associated target as base, which is compared to associated distractor, not associated target, and not associated distractor), Laterality (simple-coding matrix with central at base, which is compared to left and right), and Frontality (parietal [-0.5] vs. frontal [0.5]). LME = linear mixed effects, β = beta estimate, *SE* = standard error, *CI* = confidence interval of beta estimate. | | | | | | | |

**Table S3.11**

*Resolution of the Priming Condition, Recognition, Laterality and Frontality interaction for the N400 priming effect analysis*

| **Laterality** | **Frontality** | | **Priming Condition: Contrast Base** | **Priming Condition: Contrast Comparison** | ***t*** | ***p*** | **β** |
| --- | --- | --- | --- | --- | --- | --- | --- |
| Left | Frontal | Remem-bered | Associated Target | Associated Distractor | -13.58 | **< .001** | -0.57 |
|  |  |  | Associated Target | Not Associated Target | -11.95 | **< .001** | -0.50 |
|  |  |  | Associated Target | Not Associated Distractor | -13.68 | **< .001** | -0.57 |
|  |  |  | Associated Distractor | Not Associated Target | 1.66 | .096 | 0.07 |
|  |  |  | Associated Distractor | Not Associated Distractor | -0.05 | .960 | 0.00 |
|  |  |  | Not Associated Target | Not Associated Distractor | -1.72 | .085 | -0.07 |
|  |  | Not Remem-bered | Associated Target | Associated Distractor | 0.83 | **.028** | 0.12 |
|  |  |  | Associated Target | Not Associated Target | 2.02 | **.021** | 0.12 |
|  |  |  | Associated Target | Not Associated Distractor | 2.07 | **.004** | 0.15 |
|  |  |  | Associated Distractor | Not Associated Target | 1.19 | .233 | 0.05 |
|  |  |  | Associated Distractor | Not Associated Distractor | 1.25 | .212 | 0.05 |
|  |  |  | Not Associated Target | Not Associated Distractor | 0.06 | .956 | 0.00 |
|  | Parietal | Remem-bered | Associated Target | Associated Distractor | -13.90 | **< .001** | -0.57 |
|  |  |  | Associated Target | Not Associated Target | -13.52 | **< .001** | -0.56 |
|  |  |  | Associated Target | Not Associated Distractor | -17.04 | **< .001** | -0.70 |
|  |  |  | Associated Distractor | Not Associated Target | 0.39 | .693 | 0.02 |
|  |  |  | Associated Distractor | Not Associated Distractor | -3.08 | **.002** | -0.13 |
|  |  |  | Not Associated Target | Not Associated Distractor | -3.48 | **< .001** | -0.14 |
|  |  | Not Remem-bered | Associated Target | Associated Distractor | 1.32 | .187 | 0.06 |
|  |  |  | Associated Target | Not Associated Target | -5.01 | **< .001** | -0.22 |
|  |  |  | Associated Target | Not Associated Distractor | -4.38 | **< .001** | -0.19 |
|  |  |  | Associated Distractor | Not Associated Target | -6.33 | **< .001** | -0.28 |
|  |  |  | Associated Distractor | Not Associated Distractor | -5.70 | **< .001** | -0.25 |
|  |  |  | Not Associated Target | Not Associated Distractor | 0.63 | .532 | 0.03 |

| **Laterality** | **Frontality** |  | **Priming Condition: Contrast Base** | **Priming Condition: Contrast Comparison** | ***t*** | ***p*** | **β** |
| --- | --- | --- | --- | --- | --- | --- | --- |
| Central | Frontal | Remem-bered | Associated Target | Associated Distractor | -32.83 | **< .001** | -1.37 |
|  |  |  | Associated Target | Not Associated Target | -35.90 | **< .001** | -1.49 |
|  |  |  | Associated Target | Not Associated Distractor | -37.22 | **< .001** | -1.55 |
|  |  |  | Associated Distractor | Not Associated Target | -2.91 | **.004** | -0.12 |
|  |  |  | Associated Distractor | Not Associated Distractor | -4.20 | **< .001** | -0.17 |
|  |  |  | Not Associated Target | Not Associated Distractor | -1.30 | .193 | -0.05 |
|  |  | Not Remem-bered | Associated Target | Associated Distractor | -2.18 | **.029** | -0.10 |
|  |  |  | Associated Target | Not Associated Target | 0.67 | .504 | 0.03 |
|  |  |  | Associated Target | Not Associated Distractor | 0.63 | .527 | 0.03 |
|  |  |  | Associated Distractor | Not Associated Target | 2.84 | **.005** | 0.13 |
|  |  |  | Associated Distractor | Not Associated Distractor | 2.81 | **.005** | 0.12 |
|  |  |  | Not Associated Target | Not Associated Distractor | -0.04 | .972 | 0.00 |
|  | Parietal | Remem-bered | Associated Target | Associated Distractor | -10.72 | **< .001** | -0.62 |
|  |  |  | Associated Target | Not Associated Target | -9.82 | **< .001** | -0.57 |
|  |  |  | Associated Target | Not Associated Distractor | -15.73 | **< .001** | -0.92 |
|  |  |  | Associated Distractor | Not Associated Target | 0.91 | .362 | 0.05 |
|  |  |  | Associated Distractor | Not Associated Distractor | -5.00 | **< .001** | -0.29 |
|  |  |  | Not Associated Target | Not Associated Distractor | -5.91 | **< .001** | -0.34 |
|  |  | Not Remem-bered | Associated Target | Associated Distractor | 1.15 | .250 | 0.07 |
|  |  |  | Associated Target | Not Associated Target | -1.55 | .121 | -0.10 |
|  |  |  | Associated Target | Not Associated Distractor | -2.74 | **.006** | -0.17 |
|  |  |  | Associated Distractor | Not Associated Target | -2.71 | **.007** | -0.17 |
|  |  |  | Associated Distractor | Not Associated Distractor | -3.89 | **< .001** | -0.24 |
|  |  |  | Not Associated Target | Not Associated Distractor | -1.20 | .231 | -0.07 |

| **Laterality** | **Frontality** |  | **Priming Condition: Contrast Base** | **Priming Condition: Contrast Comparison** | ***t*** | ***p*** | **β** |
| --- | --- | --- | --- | --- | --- | --- | --- |
| Right | Frontal | Remem-bered | Associated Target | Associated Distractor | -25.82 | **< .001** | -1.08 |
|  |  |  | Associated Target | Not Associated Target | -28.02 | **< .001** | -1.17 |
|  |  |  | Associated Target | Not Associated Distractor | -26.28 | **< .001** | -1.09 |
|  |  |  | Associated Distractor | Not Associated Target | -2.14 | **.032** | -0.09 |
|  |  |  | Associated Distractor | Not Associated Distractor | -0.36 | .717 | -0.02 |
|  |  |  | Not Associated Target | Not Associated Distractor | 1.79 | .074 | 0.07 |
|  |  | Not Remem-bered | Associated Target | Associated Distractor | -0.67 | .501 | -0.03 |
|  |  |  | Associated Target | Not Associated Target | 0.02 | .988 | 0.00 |
|  |  |  | Associated Target | Not Associated Distractor | -4.62 | **< .001** | -0.20 |
|  |  |  | Associated Distractor | Not Associated Target | 0.69 | .491 | 0.03 |
|  |  |  | Associated Distractor | Not Associated Distractor | -3.96 | **< .001** | -0.17 |
|  |  |  | Not Associated Target | Not Associated Distractor | -4.64 | **< .001** | -0.21 |
|  | Parietal | Remem-bered | Associated Target | Associated Distractor | -13.97 | **< .001** | -0.57 |
|  |  |  | Associated Target | Not Associated Target | -17.70 | **< .001** | -0.73 |
|  |  |  | Associated Target | Not Associated Distractor | -18.89 | **< .001** | -0.77 |
|  |  |  | Associated Distractor | Not Associated Target | -3.70 | **< .001** | -0.15 |
|  |  |  | Associated Distractor | Not Associated Distractor | -4.88 | **< .001** | -0.20 |
|  |  |  | Not Associated Target | Not Associated Distractor | -1.18 | .237 | -0.05 |
|  |  | Not Remem-bered | Associated Target | Associated Distractor | -1.24 | .214 | -0.05 |
|  |  |  | Associated Target | Not Associated Target | -2.54 | **.011** | -0.11 |
|  |  |  | Associated Target | Not Associated Distractor | -3.90 | **< .001** | -0.17 |
|  |  |  | Associated Distractor | Not Associated Target | -1.30 | .192 | -0.06 |
|  |  |  | Associated Distractor | Not Associated Distractor | -2.66 | **.008** | -0.12 |
|  |  |  | Not Associated Target | Not Associated Distractor | -1.36 | .174 | -0.06 |

***Prediction of Association Strength measured by N400 amplitude***

**FRN-N400.**

**Table S3.12**

*Number of datapoints per participant and condition for the N400-FRN analysis*

| Feedback Timing | Priming Condition | Feedback Valence | *M* | *SD* | *Min* | *Max* |
| --- | --- | --- | --- | --- | --- | --- |
| Immediate Feedback | Associated Target | Positive Feedback | 304.55 | 59.88 | 178 | 420 |
|  |  | Negative Feedback | 158.09 | 47.18 | 58 | 246 |
|  | Unrelated | Positive Feedback | 920.94 | 179.92 | 523 | 1283 |
|  |  | Negative Feedback | 471.61 | 133.30 | 189 | 734 |
| Delayed Feedback | Associated Target | Positive Feedback | 345.06 | 59.90 | 254 | 474 |
|  |  | Negative Feedback | 137.21 | 43.22 | 74 | 232 |
|  | Unrelated | Positive Feedback | 1027.70 | 173.93 | 754 | 1440 |
|  |  | Negative Feedback | 411.21 | 129.98 | 223 | 696 |

*Note. M* = Mean, *SD* = Standard Deviation, *Min* = Minimum, *Max* = Maximum.

| **Table S3.13**  β*-Values, confidence intervals and t-test results for the LME analysis on N400 amplitude including FRN as a predictor* | | | | | | | |  |
| --- | --- | --- | --- | --- | --- | --- | --- | --- |
| **Effects** | **β** | ***SE*** | ***df*** | ***t*** | ***p*** | ***2.5% CI*** | ***97.5% CI*** | |
| Intercept | 1.31 | 0.22 | 64.00 | 5.91 | **< .001** | 0.86 | 1.77 | |
| Feedback Timing | -0.08 | 0.44 | 64.00 | -0.18 | .859 | -0.85 | 0.82 | |
| Priming Condition | 0.78 | 0.13 | 64.21 | 5.80 | **< .001** | 0.50 | 1.04 | |
| FRN | -0.20 | 0.15 | 59.45 | -1.34 | .187 | -0.50 | 0.07 | |
| Feedback Timing x Priming Condition | -0.01 | 0.27 | 64.21 | -0.03 | .974 | -0.52 | 0.55 | |
| Feedback Timing x FRN | -0.41 | 0.30 | 59.45 | -1.37 | .177 | -1.02 | 0.14 | |
| Priming Condition x FRN | -0.32 | 0.31 | 67.43 | -1.05 | .299 | -0.91 | 0.19 | |
| FRN x Feedback Valence | 0.05 | 0.25 | 80.03 | 0.18 | .854 | -0.50 | 0.55 | |
| Feedback Timing x Priming Condition x FRN | -0.54 | 0.61 | 67.43 | -0.87 | .385 | -1.72 | 0.68 | |
| Feedback Timing x FRN x Feedback Valence | 1.05 | 0.51 | 80.03 | 2.07 | **.042** | 0.05 | 2.10 | |
| Priming Condition x FRN x Feedback Valence | -0.50 | 0.38 | 46569.74 | -1.32 | .186 | -1.27 | 0.28 | |
| Feedback Timing x Priming Condition x FRN x Feedback Valence | 2.01 | 0.76 | 46569.74 | 2.63 | **.008** | 0.54 | 3.65 | |
| *Note.* The sign of the β-estimates indicates the direction of main effects for the fixed-effects predictors Feedback Timing (immediate [-0.5] vs. delayed [0.5]), FRN Amplitude (scaled and mean-centered), and Feedback Valence (nested in FRN Amplitude, negative [-0.5] vs. positive [0.5]), and Priming Condition (associated target vs. unrelated). LME = linear mixed effects, FRN = feedback-related negativity, β = beta estimate, *SE* = standard error, *CI* = confidence interval of beta estimate. | | | | | | | | |

**Figure S3.2**

*Descriptive statistics including single-trial values for the FRN-N400 model*

**
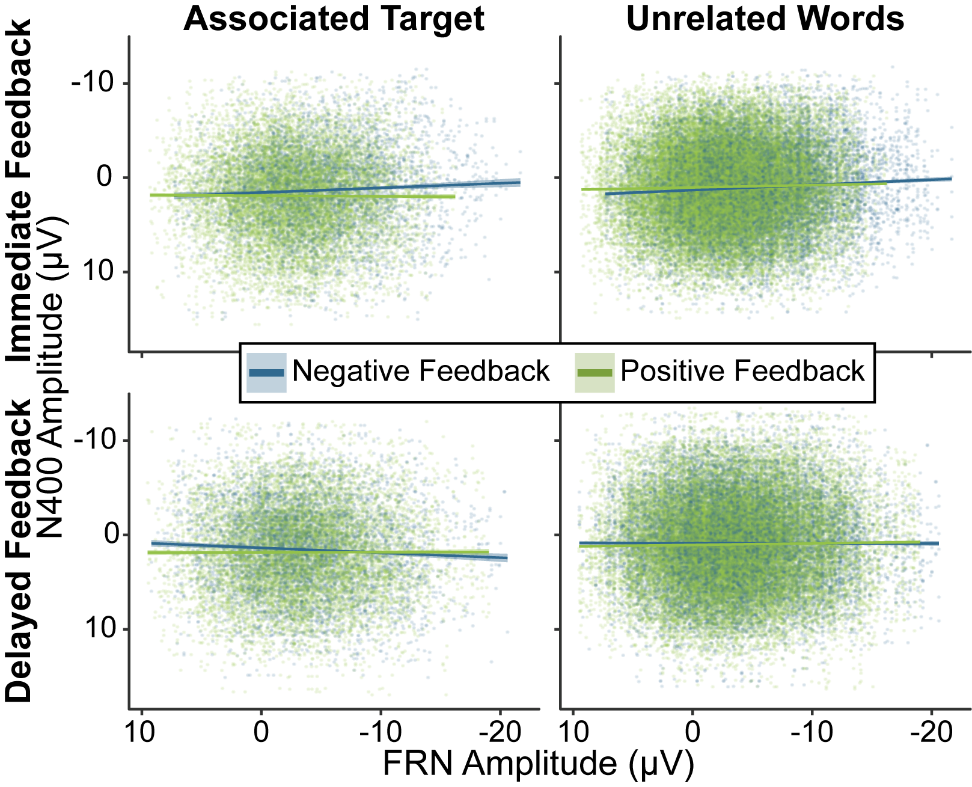
**

*Note.* Lines represent means, shaded areas around the means represent 95% confidence intervals.

**N170-N400.**

**Table S3.14**

*Number of datapoints per participant and condition for the N400-N170 analysis*

| Feedback Timing | Priming Condition | Feedback Valence | *M* | *SD* | *Min* | *Max* |
| --- | --- | --- | --- | --- | --- | --- |
| Immediate Feedback | Associated Target | Positive Feedback | 305.82 | 65.24 | 161 | 418 |
|  |  | Negative Feedback | 155.39 | 47.32 | 60 | 246 |
|  | Unrelated | Positive Feedback | 927.06 | 193.77 | 485 | 1289 |
|  |  | Negative Feedback | 462.61 | 132.36 | 193 | 734 |
| Delayed Feedback | Associated Target | Positive Feedback | 345.91 | 59.55 | 252 | 466 |
|  |  | Negative Feedback | 140.03 | 43.25 | 74 | 230 |
|  | Unrelated | Positive Feedback | 1028.45 | 172.73 | 754 | 1416 |
|  |  | Negative Feedback | 420.00 | 129.14 | 234 | 692 |

*Note. M* = Mean, *SD* = Standard Deviation, *Min* = Minimum, *Max* = Maximum.

| **Table S3.15**  β*-Values, confidence intervals and t-test results for the LME analysis on N400 amplitude including N170 as a predictor* | | | | | | | |  |
| --- | --- | --- | --- | --- | --- | --- | --- | --- |
| **Effects** | **β** | ***SE*** | ***df*** | ***t*** | ***p*** | ***2.5% CI*** | ***97.5% CI*** | |
| Intercept | 1.30 | 0.22 | 64.01 | 5.87 | **< .001** | 0.86 | 1.76 | |
| Feedback Timing | -0.03 | 0.44 | 64.01 | -0.08 | .938 | -0.93 | 0.85 | |
| Priming Condition | 0.76 | 0.14 | 63.91 | 5.60 | **< .001** | 0.48 | 1.03 | |
| N170 | -0.30 | 0.19 | 61.40 | -1.57 | .122 | -0.67 | 0.10 | |
| Feedback Timing x Priming Condition | 0.04 | 0.27 | 63.91 | 0.16 | .876 | -0.46 | 0.60 | |
| Feedback Timing x N170 | 0.41 | 0.38 | 61.40 | 1.09 | .280 | -0.25 | 1.14 | |
| Priming Condition x N170 | 0.33 | 0.37 | 64.95 | 0.90 | .372 | -0.39 | 1.07 | |
| N170 x Feedback Valence | 1.02 | 0.31 | 88.08 | 3.33 | **.001** | 0.37 | 1.60 | |
| Feedback Timing x Priming Condition x N170 | -0.11 | 0.73 | 64.95 | -0.14 | .885 | -1.54 | 1.46 | |
| Feedback Timing x N170 x Feedback Valence | 0.03 | 0.61 | 88.08 | 0.04 | .967 | -1.22 | 1.33 | |
| Priming Condition x N170 x Feedback Valence | 0.43 | 0.41 | 58538.74 | 1.05 | .294 | -0.36 | 1.20 | |
| Feedback Timing x Priming Condition x N170 x Feedback Valence | 1.06 | 0.83 | 58538.74 | 1.28 | .202 | -0.67 | 2.83 | |
| *Note.* The sign of the β-estimates indicates the direction of main effects for the fixed-effects predictors Feedback Timing (immediate [-0.5] vs. delayed [0.5]), FRN Amplitude (scaled and mean-centered), and Feedback Valence (nested in N170 Amplitude, negative [-0.5] vs. positive [0.5]), and Priming Condition (associated target vs. unrelated). LME = linear mixed effects, β = beta estimate, *SE* = standard error, *CI* = confidence interval of beta estimate. | | | | | | | | |

**Figure S3.3**

*Descriptive statistics including single-trial values for the N170-N400 model*


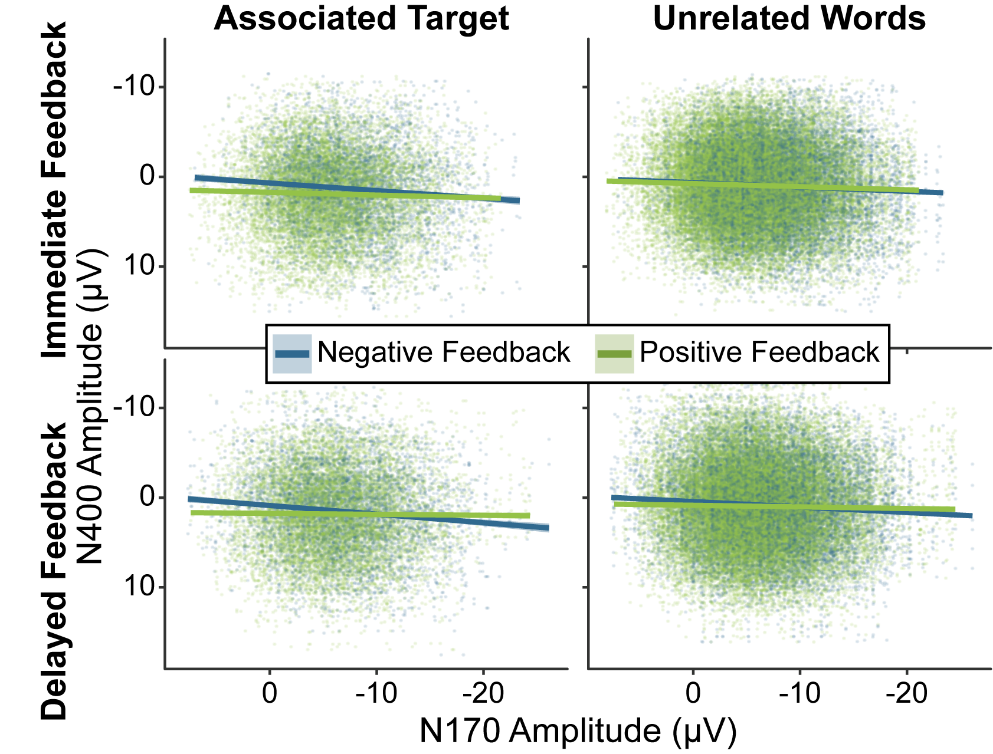


*Note.* Lines represent means, shaded areas around the means represent 95% confidence intervals.

**Section S4: Investigating the Null-Effect of FRN on Free Recall Performance**

As we found no significant effect of FRN amplitude on free recall performance (see Results section), we subsequently compared models to determine a Bayes Factor for the effect.

We determined a baseline model not containing any fixed effects including the FRN:

| $Free Recall Accuracy \sim Feedback Timing + (1 + FRN + Feedback Valence:FRN\vert Subject)$ | (S4.1) |
| --- | --- |

And compared this to our previously determined final FRN-Recall model:

| $Free Recall Accuracy \sim FRN * Feedback Timing + Feedback Valence:FRN * Feedback Timing + (1 + FRN + Feedback Valence:FRN\vert Subject)$ | (S4.2) |
| --- | --- |

The baseline model resulted in a Bayes Information Criterion (BIC) of 17929 [BIC_0_], the FRN-Recall model in a BIC of 17960 [BIC_1_]. To determine the Bayes Factor, we used the following formula:

| $B_{10} = e^{({BIC}_{0}-{BIC}_{1})/2}$ | (S4.3) |
| --- | --- |

The Bayes Factor B_10_ of including FRN as predictor thus was B_10_ < 0.001. As a rule of thumb, if Bayes Factors are below $\frac{1}{100}$, it is considered extreme evidence for the null hypothesis, i.e., extreme evidence that the effect is non-existent (Lee & Wagenmakers, 2014).

**Section S5: Exploratory Analyses on Parietal N400**

We used the same fixed effects to analyse the posterior central N400 (i.e., the N400 at electrode site Pz) as we did to investigate the frontocentral N400 (i.e., the N400 at electrode sites FCz and Fz). Random effects were determined and outlier residuals removed using the above-described procedure.

**FRN-N400 posterior.** The formula used for the FRN-N400 posterior model was as follows:

| $Posterior N400 Amplitude \sim Feedback Timing * Priming Condition * FRN + Feedback Valence:FRN * Feedback Timing * Priming Condition + (1 + Priming Condition+ FRN + FRN:Feedback Valence \vert Subject)$ | (S5.1) |
| --- | --- |

We found no significant main or interaction effect of FRN. See additional inferential statistics in Table S5.1.

| **Table S5.1**  β*-Values, confidence intervals and t-test results for the LME analysis on posterior N400 amplitude including FRN as a predictor* | | | | | | | |  |
| --- | --- | --- | --- | --- | --- | --- | --- | --- |
| **Effects** | **β** | ***SE*** | ***df*** | ***t*** | ***p*** | ***2.5% CI*** | ***97.5% CI*** | |
| Intercept | 0.64 | 0.25 | 63.94 | 2.62 | **.011** | 0.15 | 1.14 | |
| Feedback Timing | 0.29 | 0.49 | 63.94 | 0.60 | .553 | -0.64 | 1.29 | |
| Priming Condition | 0.35 | 0.11 | 63.27 | 3.09 | **.003** | 0.14 | 0.58 | |
| FRN | 0.18 | 0.15 | 87.36 | 1.21 | .230 | -0.09 | 0.45 | |
| Feedback Timing x Priming Condition | -0.07 | 0.23 | 63.27 | -0.32 | .747 | -0.54 | 0.35 | |
| Feedback Timing x FRN | -0.11 | 0.29 | 87.36 | -0.36 | .718 | -0.67 | 0.47 | |
| Priming Condition x FRN | 0.12 | 0.22 | 49267.66 | 0.53 | .597 | -0.29 | 0.58 | |
| FRN x Feedback Valence | 0.11 | 0.28 | 84.49 | 0.38 | .702 | -0.41 | 0.66 | |
| Feedback Timing x Priming Condition x FRN | -0.81 | 0.45 | 49267.66 | -1.80 | .072 | -1.81 | -0.02 | |
| Feedback Timing x FRN x Feedback Valence | -0.92 | 0.57 | 84.49 | -1.62 | .108 | -2.09 | 0.32 | |
| Priming Condition x FRN x Feedback Valence | 0.33 | 0.42 | 62015.66 | 0.77 | .441 | -0.53 | 1.19 | |
| Feedback Timing x Priming Condition x FRN x Feedback Valence | -0.71 | 0.85 | 62015.66 | -0.84 | .402 | -2.19 | 0.85 | |
| *Note.* The sign of the β-estimates indicates the direction of main effects for the fixed-effects predictors Feedback Timing (immediate [-0.5] vs. delayed [0.5]), FRN Amplitude (scaled and mean-centered), and Feedback Valence (nested in FRN Amplitude, negative [-0.5] vs. positive [0.5]), and Priming Condition (associated target [0.5] vs. unrelated [-0.5]). LME = linear mixed effects, FRN = feedback-related negativity, β = beta estimate, *SE* = standard error, *CI* = confidence interval of beta estimate. | | | | | | | | |

**N170-N400 posterior.** The formula for the N170-N400 posterior model was as follows:

| $Posterior N400 Amplitude \sim Feedback Timing * Priming Condition * FRN + Feedback Valence:FRN * Feedback Timing * Priming Condition + (1 + Priming Condition+ N170 + N170:Feedback Valence \vert Subject)$ | (S5.2) |
| --- | --- |

We found a main effect of N170, *F*(1,80.82) = 6.89, *p* = .010, β = 0.53. The more negative N170 amplitudes were during learning, the more negative were posterior N400 amplitudes in the primed recognition task. We also found an interaction between Feedback Timing, Priming Condition, N170 and Feedback Valence, *F*(1,62184.79) = 6.45, *p* = .011. Resolving the interaction, a three-way interaction between Priming Condition, N170 and Feedback Valence emerged only for delayed feedback, *F*(1,62163.77) = 5.10, *p* = .024 (*p* = .176 for immediate feedback). Resolving further by Feedback Valence, a Priming Condition by N170 interaction emerged only for negative feedback, *F*(1,48806.56) = 4.95, *p* = .026 (*p* = .563 for positive feedback). Finally, a N170 effect emerged for delayed negative feedback in the associated target condition, *F*(1,282.29) = 4.21, *p* = .041, β = 1.17, but not in the unrelated condition (*p* = .849), with larger N170 amplitudes predicting larger N400 amplitudes. See additional inferential statistics in Table S5.2.

| **Table S5.2**  β*-Values, confidence intervals and t-test results for the LME analysis on posterior N400 amplitude including N170 as a predictor* | | | | | | |  | |
| --- | --- | --- | --- | --- | --- | --- | --- | --- |
| **Effects** | **β** | ***SE*** | ***df*** | ***t*** | ***p*** | ***2.5% CI*** | | ***97.5% CI*** |
| Intercept | 0.65 | 0.24 | 63.95 | 2.64 | **.010** | 0.18 | | 1.12 |
| Feedback Timing | 0.27 | 0.49 | 63.95 | 0.56 | .580 | -0.64 | | 1.19 |
| Priming Condition | 0.38 | 0.11 | 63.22 | 3.32 | **.002** | 0.16 | | 0.58 |
| N170 | 0.53 | 0.20 | 80.82 | 2.62 | **.010** | 0.06 | | 0.92 |
| Feedback Timing x Priming Condition | -0.04 | 0.23 | 63.22 | -0.17 | .868 | -0.51 | | 0.41 |
| Feedback Timing x N170 | -0.03 | 0.40 | 80.82 | -0.09 | .931 | -0.83 | | 0.78 |
| Priming Condition x N170 | 0.53 | 0.28 | 26883.15 | 1.92 | .055 | -0.03 | | 1.09 |
| N170 x Feedback Valence | 0.00 | 0.31 | 80.68 | -0.02 | .988 | -0.61 | | 0.66 |
| Feedback Timing x Priming Condition x N170 | -0.07 | 0.55 | 26883.15 | -0.13 | .897 | -1.28 | | 0.95 |
| Feedback Timing x N170 x Feedback Valence | -0.12 | 0.62 | 80.68 | -0.19 | .849 | -1.20 | | 1.25 |
| Priming Condition x N170 x Feedback Valence | -0.28 | 0.48 | 62184.79 | -0.59 | .557 | -1.17 | | 0.61 |
| Feedback Timing x Priming Condition x N170 x Feedback Valence | -2.45 | 0.96 | 62184.79 | -2.54 | **.011** | -4.20 | | -0.54 |
| *Note.* The sign of the β-estimates indicates the direction of main effects for the fixed-effects predictors Feedback Timing (immediate [-0.5] vs. delayed [0.5]), N170 Amplitude (scaled and mean-centered), and Feedback Valence (nested in FRN Amplitude, negative [-0.5] vs. positive [0.5]), and Priming Condition (associated target [0.5] vs. unrelated [-0.5]). LME = linear mixed effects, β = beta estimate, *SE* = standard error, *CI* = confidence interval of beta estimate. | | | | | | | | |

**Section S6: Post-Hoc Analyses on the P300**

First, we set up a control analysis to examine the modulatory effects of Feedback Valence (and Feedback Timing) on the feedback-locked P300. Second, we set up three analyses to test whether behavioral recall, recognition performance and N400 amplitudes can be predicted from feedback-locked P300 during learning. To extract the P300, we used the same preprocessed ERP segments also used to extract the FRN and the N170. We pooled the signal across the central electrodes Fz, Cz, and Pz, and calculated the area mean between 350 and 450 ms for each segment. These values served as the dependent variable in the P300 control analysis and as predictor in the recall, recognition and N400 models. The number of included datapoints for the analyses are reported in Table S6.1. Detailed description of all the analyses and the results are reported in the following.

**Table S6.1**

*Number of datapoints per participant and condition for the P300 control analysis, P300-Recall analysis and P300-Recognition analysis*

| Feedback Timing | Feedback Valence | *M* | *SD* | *Min* | *Max* |
| --- | --- | --- | --- | --- | --- |
| Immediate Feedback | Positive Feedback | 185.97 | 29.39 | 127 | 238 |
|  | Negative Feedback | 74.12 | 21.66 | 37 | 118 |
| Delayed Feedback | Positive Feedback | 173.97 | 27.87 | 117 | 227 |
|  | Negative Feedback | 88.36 | 24.13 | 34 | 127 |

*Note. M* = Mean, *SD* = Standard Deviation, *Min* = Minimum, *Max* = Maximum

**Control Analysis**

Figure S6.1 shows grand average waveforms and topographies of the P300 for immediate and delayed positive and negative feedback during learning, as well as mean, standard deviations and distributions (violin plots) of the conditions included in the model specified for the control analysis.

The control analysis model for the P300 included the fixed effects Feedback Valence (positive [0.5], negative [-0.5]), and Feedback Timing (immediate [-0.5], delayed [0.5]), as well as their interaction. The model also included random intercepts by participant and random slopes of Feedback Valence by participant. This analysis revealed a significant main effect of Feedback Valence, *F*(1,62.80) = 78.39, *p* < .001, β = -1.19, with a larger P300 for negative compared to positive feedback. We also found a significant Feedback Timing by Feedback Valence interaction, *F*(1,62.80) = 5.74, β = .020. For both delayed feedback, *F*(1,61.87) = 21.01, *p* < .001, β = -0.87, and immediate feedback, *F*(1,63.74) = 62.81, *p* < .001, β = -1.52, amplitudes were more pronounced for negative feedback, but this effect was stronger for immediate feedback. No main effect of Feedback Timing emerged (*p* = .250). Additional statistical results are reported in Table S6.2.

| **Table S6.2**  β*-Values, confidence intervals and t-test results for the LME control analysis on P300 amplitude* | | | | | | | |  |
| --- | --- | --- | --- | --- | --- | --- | --- | --- |
| **Effects** | **β** | ***SE*** | ***df*** | ***t*** | ***p*** | ***2.5% CI*** | ***97.5% CI*** | |
| Intercept | 2.79 | 0.21 | 63.99 | 13.37 | **< .001** | 2.40 | 3.18 | |
| Feedback Timing | 0.48 | 0.42 | 63.99 | 1.16 | .250 | -0.30 | 1.33 | |
| Feedback Valence | -1.19 | 0.13 | 62.80 | -8.85 | **< .001** | -1.45 | -0.93 | |
| Feedback Timing x Feedback Valence | 0.65 | 0.27 | 62.80 | 2.39 | **.020** | 0.12 | 1.18 | |
| *Note.* The sign of the β-estimates indicates the direction of main effects for the fixed-effects predictors Feedback Timing (immediate [-0.5], delayed [0.5]), and Feedback Valence (negative [-0.5], positive [0.5]). LME = linear mixed effects, β = beta estimate, *SE* = standard error, *CI* = confidence interval of beta estimate. | | | | | | | | |

**Figure S6.1**

*Feedback-locked grand average ERP waveforms, topographies and control model descriptive results for the P300 area extraction*


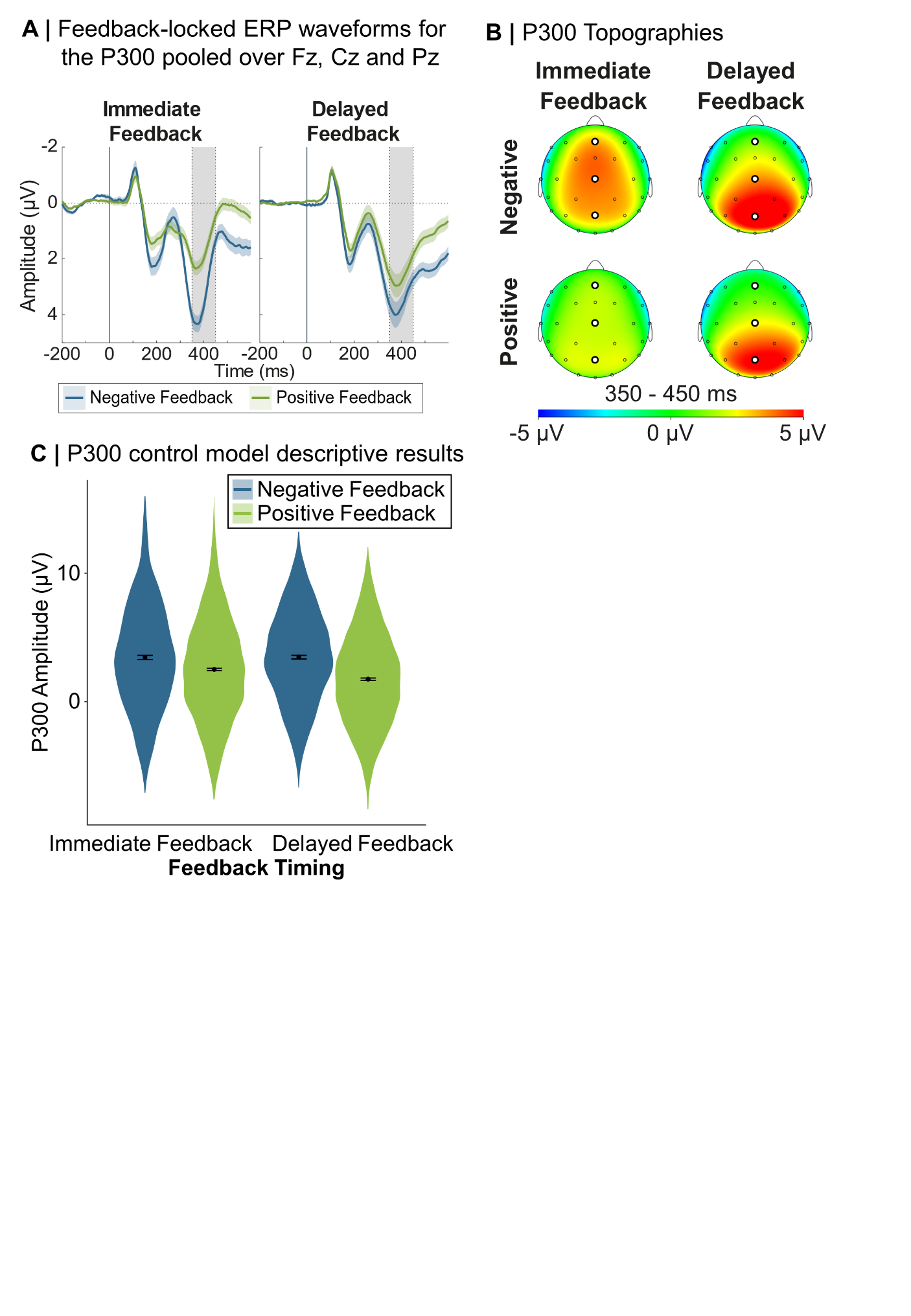


Note. Error margins around the grand averages *(n* = 33 in both Feedback Delay conditions) represent ± 1 standard error. Gray areas represent the time window in which the P300 area means were extracted. Error margins in subplot C represent 95% confidence intervals.

**Prediction of Behavioral Recall Performance**

The P300-Recall model was determined as:

| $Free Recall Accuracy \sim Feedback Timing * P300 + Feedback Valence : P300 * Feedback Timing + (1 + P300 + P300 : Feedback Valence \vert Subject)$ | (S6.1) |
| --- | --- |

We found a significant main effect of P300 Amplitude on free recall accuracy, *z* = -6.36, *p* < .001, β = -1.21. The more positive the P300, the lower the recall accuracy. This main effect was further explained by an interaction between P300 Amplitude and Feedback Timing, *z* = 2.92, *p* = .003, and a three-way interaction between P300, Feedback Timing and Feedback Valence. The interaction between Feedback Valence and P300 Amplitude emerged for immediate feedback, *z* = -1.99, *p* = .047, but not for delayed feedback (*p* = .465). The described P300 Amplitude effect was more pronounced for immediate positive feedback, *z* = -7.78, *p* < .001, β = -2.23, than immediate negative feedback, *z* = -3.01, *p* = .003, β = -1.27. No other effects were significant (all *p* ≤ .340). Additional statistical results are reported in Table S6.3. Model estimates for the P300-Recall are displayed in Figure S6.2.

| **Table S6.3**  β*-Values, confidence intervals and t-test results for the GLME analysis on free recall accuracy including P300 as predictor* | | | | | | |  |
| --- | --- | --- | --- | --- | --- | --- | --- |
| **Effects** | **β** | ***SE*** | ***z*** | ***p*** | ***2.5% CI*** | ***97.5% CI*** | |
| Intercept | -0.43 | 0.23 | -1.90 | .058 | -0.88 | 0.00 | |
| Feedback Timing | -0.30 | 0.45 | -0.67 | .502 | -1.14 | 0.63 | |
| P300 Amplitude | -1.21 | 0.19 | -6.36 | **< .001** | -1.56 | -0.81 | |
| Feedback Timing x P300 Amplitude | 1.08 | 0.37 | 2.92 | **.003** | 0.34 | 1.91 | |
| P300 Amplitude x Feedback Valence | -0.32 | 0.33 | -0.95 | .340 | -1.05 | 0.40 | |
| Feedback Timing x P300 Amplitude x Feedback Valence | 1.28 | 0.65 | 1.97 | **.049** | -0.28 | 2.66 | |
| *Note.* The sign of the β-estimates indicates the direction of main effects for the fixed-effects predictors Feedback Timing (immediate [-0.5], delayed [0.5]), P300 Amplitude (scaled and mean-centered), and Feedback Valence (nested in P300 Amplitude, negative [-0.5], positive [0.5]). GLME: generalized linear mixed effects, β = beta estimate, *SE* = standard error, *CI* = confidence interval of beta estimate. | | | | | | | |

**Figure S6.2**

*Model estimates for models in which the P300 A. predicted Recall, and B. predicted Recognition*

*
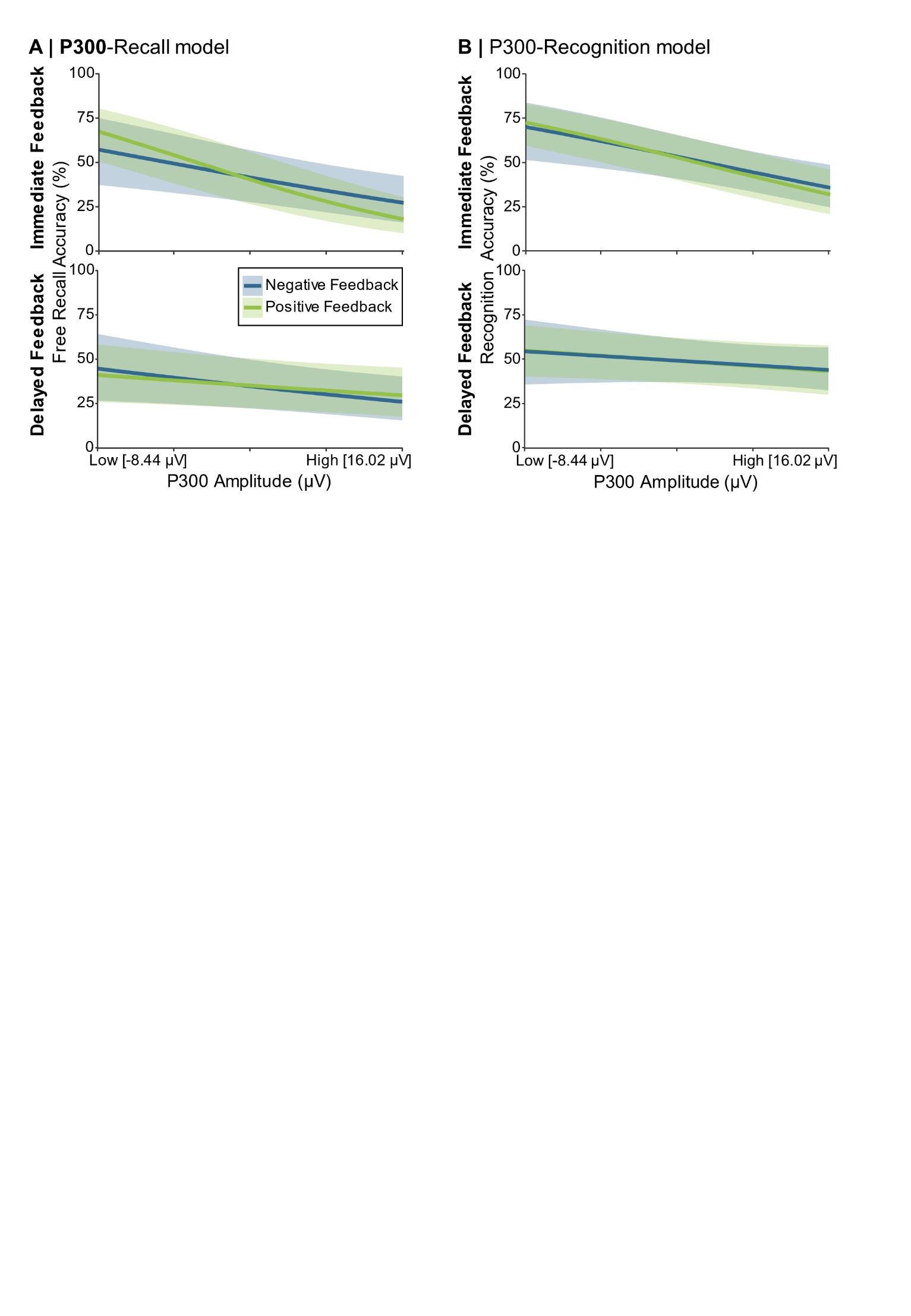
*

*Note.* The blue and green lines depict the GLME predictions based on fitted β weights and intercepts. Error margins represent 95% confidence intervals of the model predictions. Amplitude of the P300 as predictor on the x-axes are depicted from least to most positive (i.e., low to high amplitude).

**Prediction of Behavioral Recognition Performance**

The P300-Recognition model was determined as:

| $Recognition Accuracy \sim Feedback Timing * P300 + Feedback Valence : P300 * Feedback Timing + (1 + P300 + P300 : Feedback Valence \vert Subject)$ | (S6.2) |
| --- | --- |

We found a significant main effect of P300 Amplitude, *z* = -5.00, *p* < .001, β = -1.01. The more positive the P300, the lower the recognition accuracy. This was qualified by a Feedback Timing by P300 Amplitude interaction, *z* = 2.80, *p* = .005. The aforementioned effect was significant only for immediate feedback, *z* = -5.43, *p* < .001, β = -1.58, but not for delayed (*p* = .111). No other effects were significant (*p* ≥ .570). Additional statistical results are reported in Table S6.4. Model estimates for the P300-Recall are displayed in Figure S6.2.

| **Table S6.4**  β*-Values, confidence intervals and t-test results for the GLME analysis on recognition accuracy including P300 as predictor* | | | | | | |  |
| --- | --- | --- | --- | --- | --- | --- | --- |
| **Effects** | **β** | ***SE*** | ***z*** | ***p*** | ***2.5% CI*** | ***97.5% CI*** | |
| Intercept | 0.10 | 0.19 | 0.51 | .612 | -0.29 | 0.48 | |
| Feedback Timing | -0.21 | 0.38 | -0.57 | .570 | -0.93 | 0.53 | |
| P300 Amplitude | -1.01 | 0.20 | -5.00 | **< .001** | -1.39 | -0.62 | |
| Feedback Timing x P300 Amplitude | 1.13 | 0.40 | 2.80 | **.005** | 0.38 | 1.96 | |
| P300 Amplitude x Feedback Valence | -0.18 | 0.34 | -0.52 | .606 | -0.90 | 0.49 | |
| Feedback Timing x P300 Amplitude x Feedback Valence | 0.24 | 0.68 | 0.36 | .721 | -1.06 | 1.62 | |
| *Note.* The sign of the β-estimates indicates the direction of main effects for the fixed-effects predictors Feedback Timing (immediate [-0.5], delayed [0.5]), P300 Amplitude (scaled and mean-centered), and Feedback Valence (nested in P300 Amplitude, negative [-0.5], positive [0.5]). GLME: generalized linear mixed effects, β = beta estimate, *SE* = standard error, *CI* = confidence interval of beta estimate. | | | | | | | |

**Prediction of Association Strength measured by N400 amplitude**

The P300-N400 model was determined as:

| $N400 Amplitude \sim Feedback Timing * Priming Condition * P300 + Feedback Valence:P300 * Feedback Timing * Priming Condition + (1 + Priming Condition * P300+ P300:Feedback Valence \vert Subject)$ | (S6.3) |
| --- | --- |

Besides a significant effect of Priming Condition, *F*(1,63.96) = 31.05, *p* < .001, β = 0.76, a Feedback Timing by P300 interaction emerged, *F*(1,67.36) = 4.05, *p* = .048. However, the resolution of this interaction did not yield a conclusive pattern: neither resolving by P300 (both *p* ≥ .728) nor by Feedback Timing (both *p* ≥ .115) yielded significant main effects of the respective other factor at any level of the factor by which was resolved. Descriptively, but not statistically significant, higher P300 amplitudes led to reduced N400 amplitudes in immediate feedback (*p* = .215, β = 0.31) but increased N400 amplitudes in delayed feedback (*p* = .115, β = -0.39). No other main or interaction effects were significant (all *p* ≥ .153). Additional statistical results are reported in Table S6.5. Model estimates for the P300-N400 model are displayed in Figure S6.3.

| **Table S6.5**  β*-Values, confidence intervals and t-test results for the LME analysis on N400 amplitude including P300 as a predictor* | | | | | | | |  |
| --- | --- | --- | --- | --- | --- | --- | --- | --- |
| **Effects** | **β** | ***SE*** | ***df*** | ***t*** | ***p*** | ***2.5% CI*** | ***97.5% CI*** | |
| Intercept | 1.33 | 0.22 | 64.01 | 5.97 | **< .001** | 0.93 | 1.78 | |
| Feedback Timing | -0.05 | 0.45 | 64.01 | -0.12 | .904 | -0.97 | 0.83 | |
| Priming Condition | 0.76 | 0.14 | 63.96 | 5.57 | **< .001** | 0.48 | 1.02 | |
| P300 | -0.04 | 0.17 | 67.36 | -0.22 | .826 | -0.41 | 0.29 | |
| Feedback Timing x Priming Condition | 0.04 | 0.27 | 63.96 | 0.15 | .878 | -0.47 | 0.58 | |
| Feedback Timing x P300 | -0.70 | 0.35 | 67.36 | -2.01 | **.048** | -1.42 | -0.07 | |
| Priming Condition x P300 | -0.56 | 0.39 | 71.45 | -1.44 | .153 | -1.41 | 0.21 | |
| P300 x Feedback Valence | 0.20 | 0.33 | 72.85 | 0.60 | .552 | -0.48 | 0.79 | |
| Feedback Timing x Priming Condition x P300 | -0.56 | 0.78 | 71.45 | -0.71 | .477 | -2.14 | 1.06 | |
| Feedback Timing x P300 x Feedback Valence | 0.33 | 0.66 | 72.85 | 0.50 | .618 | -1.06 | 1.68 | |
| Priming Condition x P300 x Feedback Valence | -0.31 | 0.41 | 92757.02 | -0.75 | .452 | -1.16 | 0.53 | |
| Feedback Timing x Priming Condition x P300 x Feedback Valence | 0.06 | 0.82 | 92757.02 | 0.07 | .946 | -1.52 | 1.76 | |
| *Note.* The sign of the β-estimates indicates the direction of main effects for the fixed-effects predictors Feedback Timing (immediate [-0.5], delayed [0.5]), P300 Amplitude (scaled and mean-centered), and Feedback Valence (nested in P300 Amplitude, negative [-0.5], positive [0.5]), and Priming Condition (associated target vs. unrelated). LME = linear mixed effects, β = beta estimate, *SE* = standard error, *CI* = confidence interval of beta estimate. | | | | | | | | |

**Figure S6.3**

*Model estimates for the P300-N400 model*

*
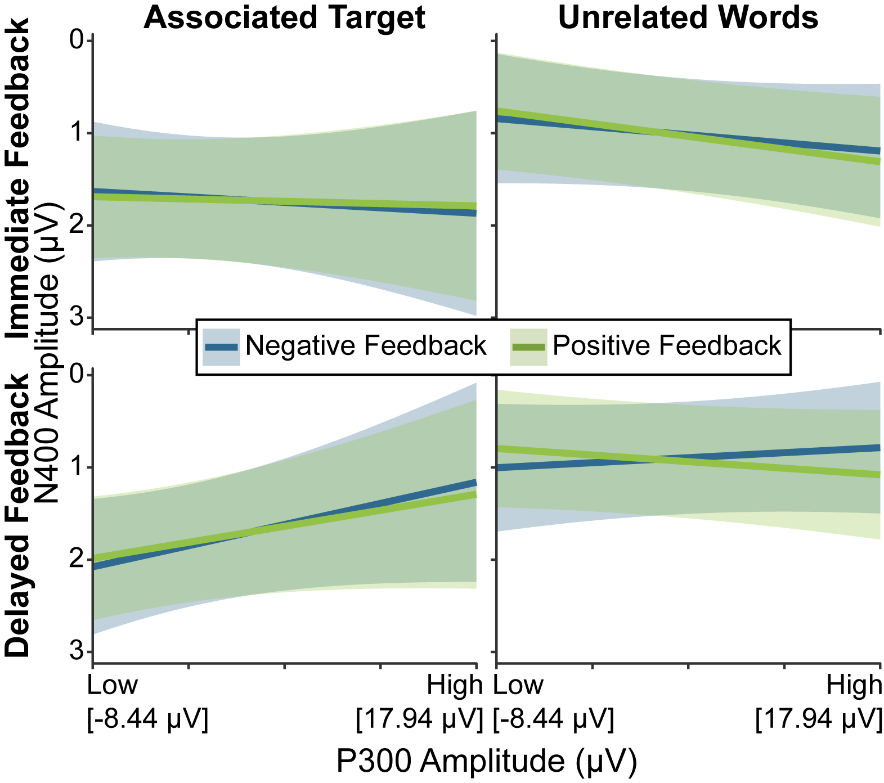
*

*Notes.* The blue and yellow lines depict the GLME predictions based on fitted β weights and intercepts. Error margins represent 95% confidence intervals of the model predictions. Amplitude of the P300 as predictor on the x-axis are depicted from least to most positive (i.e., low to high amplitude).
